# Chirality Induced in Tetraethyllead through Noncovalent Interactions with a Chiral Tag

**DOI:** 10.1021/jacs.5c21116

**Published:** 2026-02-06

**Authors:** Wenhao Sun, Steffen M. Giesen, Robert Berger, Melanie Schnell

**Affiliations:** † 28332 Deutsches Elektronen-Synchrotron DESY, Notkestr. 85, 22607 Hamburg, Germany; ‡ Fachbereich Chemie, Theoretische Chemie, 9377 Philipps-Universität Marburg, Hans-Meerwein-Str. 4, 35032 Marburg, Germany; § Institute of Physical Chemistry, Christian-Albrechts-Universität zu Kiel, Max-Eyth-Str. 1, 24118 Kiel, Germany

## Abstract

Weakly bound complexes containing lead (Pb) were studied in a supersonic jet using broadband chirped-pulse Fourier transform microwave (CP-FTMW) spectroscopy, complemented with quantum-chemical calculations. These complexes were formed from a vapor mixture of tetraethyllead (TEL) and 2-(trifluoromethyl)­oxirane (TFO), diluted in a Ne carrier gas. Theoretical isomer searches reveal 75 nearly isoenergetic isomers of the TEL-TFO dimer, all within an energy range of 1.0 kJ/mol. Rotational spectroscopy has unambiguously identified the global-minimum configuration in the ground vibrational state, including its three singly substituted ^206/207/208^Pb isotopologues. This assignment is further supported by the observation of the TEL-TFO-Ne trimer, which gives rise to six doubly substituted ^206/207/208^Pb-^20/22^Ne isotopologues in their natural abundance. The experimental data provides a valuable benchmark for assessing modern quantum-chemical methods, particularly in the treatment of relativistic effects of heavy nuclei along with noncovalent interactions. Additionally, as heavy-atom-containing chiral complexes, parity-violating effects were calculated for the TEL-TFO dimer at various levels of theory, shedding light on parity nonconservation in weakly bound complexes involving heavy nuclei.

## Graphical abstract

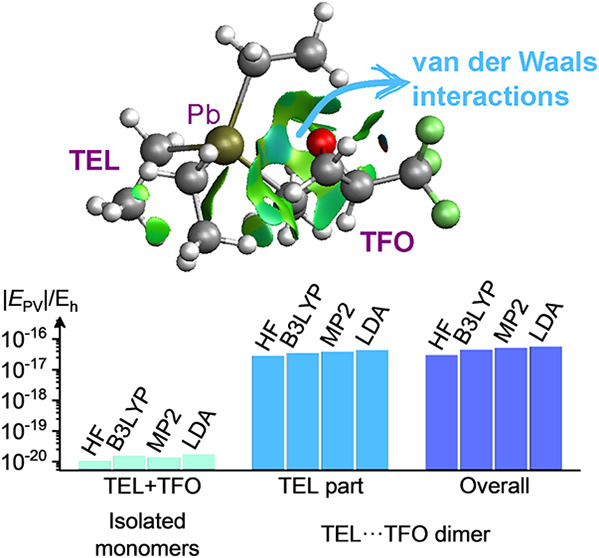

## Introduction

Chirality, from the atomic level to the molecules of life, represents a key characteristic of the intrinsic asymmetry of nature.^1^ Since the groundbreaking discovery of parity nonconservation (PNC) in atomic nuclei, electroweak parity-violating (PV) effects have also been suggested as a fundamental force responsible for various chiral behaviors in molecular physics, chemistry, and biology.^2–5^ However, the resulting energy differences (Δ*E*_pv_) between left- and right-handed enantiomers of chiral molecules are exceedingly small, posing significant challenges for their experimental detection.^6,7^ No successful observations for chiral molecular systems have been reported yet. Theoretical PNC studies indicate that PV effects scale approximately with an enhancement factor *Z* ^5^, where *Z* denotes the dominant heavy nuclear charge in the molecule.^4,8–12^ Heavy atoms in chiral molecular environments are thus highly appealing for theoretical and experimental explorations.^13–15^ Lead (_82_Pb), being the heaviest nonradioactive element,^16^ is of particular interest.

Parity-violating potentials do not only induce energy differences between enantiomers but also cause slight changes in their equilibrium structures, giving rise to small frequency shifts in rotational transitions and vibrational modes.^17,18^ The latter allows for a potential detection of PV effects through advanced precision experiments, employing ultrahigh-resolution microwave (MW) and infrared (IR) spectroscopy in combination with gas-phase molecular sources.^19^ While current MW techniques for routine applications do not yet have the resolution and precision required for establishing molecular PV frequency shifts, spectroscopic characterization of relevant molecular systems remains highly valuable.^20^ Such studies offer crucial molecular insights to guide future high-precision measurements and serve as benchmarks for theoretical models, especially with the treatment of relativistic effects.^18,21^

Several model molecules, such as heavier polyhalogenated methanes, cubanes, and transition metal compounds, have been proposed as potential molecular candidates for such high-resolution measurements and extensively studied theoretically.^14,22–24^ However, preparing enantiomer-enriched samples of these tailored molecules in the laboratory and transferring them into the gas phase for spectroscopic characterization can be a tedious and sometimes infeasible task. For this reason, gas-phase investigations of chiral molecules incorporating heavy elements, specifically those heavier than krypton (_36_Kr), have been significantly limited.^25,26^ The heaviest element explored in this context is rhenium in a chiral [CpRe­(CH_3_)­(CO)­(NO)] (Cp = η^5^-cyclopentadienyl) complex, which was successfully assessed using MW spectroscopy a decade ago.^25^ In contrast, a wide range of achiral molecules containing heavy nuclei, such as diatomic species^27–29^ and coordination complexes,^30–33^ has been more extensively studied in the gas phase using molecular spectroscopy.^34,35^ However, introducing chirality in these systems through chemical modification is challenging.

To advance experimental investigations in this field, another strategy is to generate noncovalently bound chiral complexes in a molecular jet, using heavy-atom-containing achiral molecules paired with chiral agents, which is the focus of this work. This bypasses the need for sophisticated chemical synthesis by choosing complexing components that are easy to prepare or commercially available, even in enantio-enriched form. However, unlike chemically synthesized stable molecules, the large chemical space of van der Waals complexes makes it highly demanding to determine the structures and interpret the spectra, both of which are essential to obtain the relevant arrangement of the nuclei required for further PV calculations. With recent advancements in semiempirical quantum-chemical methods within the framework of meta-dynamics, such as the GFN2-xTB tight-binding approach, these challenges are becoming more manageable.^36,37^ Nonetheless, due to the limited experimental data available, the feasibility and potential of these systems remains uncertain.

In this work, we investigated a weakly bound cluster formed from tetraethyllead (see Figure 1) and 2-(trifluoromethyl)­oxirane. The latter, being chiral, volatile, and polar, is widely used as a tagging molecule in chiral-tag microwave spectroscopy, with enantiopure samples readily accessible.^38^ To characterize the dimeric structure, we recorded its rotational spectrum using a broadband chirped-pulse Fourier transform microwave (CP-FTMW) spectrometer in combination with a supersonic jet. Quantum-chemical calculations were conducted to assist in spectral assignments. Furthermore, recent advancements in theoretical methods now allow for the evaluation of PV effects in polyatomic heavy-atom-containing compounds in weakly bound complexes. Leveraging these developments, we performed PV calculations based on the experimentally determined structure, offering insights into the emergence of PV effects in this weakly bound chiral system.

**Figure 1:**
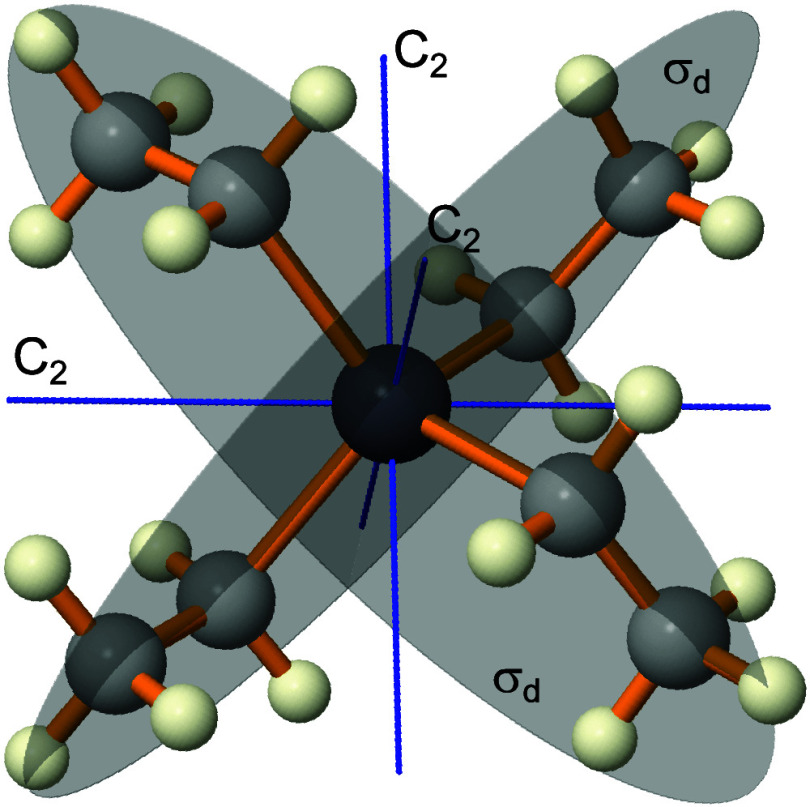
Achiral molecular structure of tetraethyllead (TEL) with *D*_2d_ point group symmetry. Symmetry elements including three *C*_2_ axes and two σ_d_ planes are depicted. The two *S*_4_ axes are not explicitly indicated, but they are aligned with the *C*_2_ axis at the intersection of the *σ*_d_ planes.

## Experimental Details

The experiment is performed with the chirped-pulse Fourier transform microwave (CP-FTMW) spectrometer COMPACT (compact-passage acquired coherence technique). Details regarding the instrument’s operation principle have been described elsewhere.^39^ 2-(Trifluoro-methyl)­oxirane and tetraethyllead, abbreviated hereinafter as TFO and TEL, are commercially available from Alfa Aesar and Gelest, with chemical purities of 97% and >95%, respectively, and are used without further purification in the experiment. The TFO sample, with a boiling point of 25–32 °C, was vaporized and diluted in neon to achieve a gas mixture with a TFO concentration of 0.2%. On the other hand, TEL, a liquid at room temperature with a melting point of −136 °C and a boiling point of 82–85 °C (at 20 mbar), was contained within a stainless-steel reservoir integrated into the solenoid nozzle (General Valve Series 9). To generate sufficient vapor, the sample reservoir was maintained at 80 °C. The generated TEL vapor was mixed with the TFO/Ne gas mixture and introduced together into the vacuum chamber of the spectrometer via supersonic expansion at a stagnation pressure of 1 bar. Weakly bound complexes were formed at the initial stage of the supersonic expansion and remained isolated during the microwave experiment due to the collision-free conditions of the cold supersonic jet.

The molecular species in the gas jet were repeatedly polarized eight times using a 4 μs-long microwave chirp covering frequencies between 2 to 8 GHz. Following each polarization interaction, a macroscopic polarization was induced within the molecular ensemble and the relaxation of the polarization was monitored as a function of time, in the form of a free induction decay (FID). The solenoid valve was pulsed at 8 Hz, resulting in an effective repetition rate of 64 Hz. A total of 4.2 × 10^6^ FID acquisitions were recorded and averaged on a fast oscilloscope using a sampling rate of 25 GSa/s. The averaged time-domain FIDs were fast Fourier transformed (FFT) to obtain the spectrum in the frequency domain, employing a Kaiser window function. The recorded duration of each FID is 40 μs, providing a spectral resolution of 25 kHz. The full-width-half-maximum (FWHM) line widths of the rotational transitions are about 60 kHz, and the frequency accuracy is approximately 10 kHz.

## Computational Details

### Isomer Search and Structure Optimizations

Initially, the binding motif of the weakly bound TEL-TFO dimer was exhaustively explored using the CREST (Conformer-Rotamer Ensemble Sampling Tool) program at the GFN2-xTB level.^36,37^ Subsequently, the preliminary structural isomers, generated within an energy window of 6.0 kJ/mol, are subjected to further optimizations at the B3LYP/def2-QZVP level of theory, in combination with the D4 dispersion corrections, using the ORCA program package Version 5.0.4.^40–42^ The def2-QZVP basis set incorporates all-electron representations for elements H, C, O, and F, while employing the Stuttgart–Dresden effective core potentials for the Pb atom.^41^ Multiple CREST–ORCA optimization cycles were conducted due to the flexible conformational space of the TEL monomer, aiming to exhaustively uncover the isomerism of the weakly bound complexes. Throughout this process, specific structural constraints, such as the Pb···O distance, were optionally applied in the CREST searches, guided by experimental assignments. Harmonic vibrational frequency calculations were performed to confirm the energy minima and provide zero-point energy (ZPE) corrections. To characterize intermolecular interactions in the dimer, a so-called noncovalent interaction (NCI) analysis and electrostatic potential (ESP) calculations were carried out using the Multiwfn program,^43^ and the results were visualized using VMD.^44^ The NCI analysis followed a procedure that has been widely used in the literature (ref. 45).

Furthermore, the unexpected detection of the TEL-TFO-Ne trimer in the microwave spectrum triggered its isomer search using the CREST–ORCA optimization workflow. For this challenging task, two sets of input structures were configured for the CREST calculations. The first set consisted of arbitrary arrangements of TEL and TFO monomers along with a Ne atom, while the second set positioned a Ne atom at various positions around the experimentally determined TEL-TFO dimer structure. In the second set, the TEL-TFO dimer was kept either constrained or relaxed during the CREST calculations. The constrained calculations assumed that incorporating Ne into the complex did not alter the geometry of the TEL-TFO dimer. Afterward, low-energy isomers were collected from the exhaustive CREST calculations for the B3LYP-D4 optimizations, with an energy threshold of 6.0 kJ/mol, consistent with the theory level used for the dimer calculations.

### Parity-Violation Calculations

At the generalized Hartree–Fock (GHF) and generalized Kohn–Sham-DFT (GKS) level, the electroweak PV effects were predicted using a quasi-relativistic (two-component) zeroth-order regular approximation approach^46,47^ to electroweak quantum chemistry,^48,49^ implemented in a modified version^50^ of Turbomole.^51^ Electron correlation effects on the post Hartree-Fock level were then included for the PV energy expectation values using two-component second-order Møller–Plesset perturbation theory (2c-MP2) gradients starting from the GHF calculations using linear response orbital transformation matrices.^18,52^ The 2c-MP2 energy and gradient calculations were implemented into *nonorth*, a program for the manipulation and analysis of (nonorthogonal) wave functions. The algorithm of choice, based on ref. 53, was extended to complex two-component orbitals and optimized for computational and memory efficiency together with an MPI/OpenMP hybrid parallelization scheme for both the occupied orbitals and basis functions. Details of the implementation will be reported elsewhere.

The equilibrium structures obtained in the previous sections were also used for the PV calculations. We used the x2c-TZVPall-2c basis set^54^ for all atoms, to maintain a balance between sufficiently steep functions for the description of the PV effects, polarization functions for electron correlation at the 2c-MP2-level and required computation time and memory in the 2c-MP2 gradient calculations. We selected a finite nuclear model with Gaussian nuclear density distribution for this numerical study, the value of Fermi’s constant was set to *G*_F_ = 2.222 49 × 10^–14^ *E*_h_ *a*_0_^3^, the value of the Weinberg parameter was set to sin^2^(θ_w_) ≈ 0.2319, and the isotopes ^208^Pb, ^19^F, ^16^O, ^12^C, and ^1^H were considered in the computation of the PV energy shift.

## Results and Discussion

### Spectral Analysis and Structural Characterization

The *D*_2d_ point group symmetry of TEL makes it nonpolar (Figure 1), therefore it cannot be directly studied using pure rotational spectroscopy. In order to put the element Pb in a chiral molecular environment, a chiral complexing partner, TFO, is used to form weakly bound complexes with TEL potentially through what has been referred to as tetrel bonding interactions, in which the electronegative oxygen atom in TFO is preferably attracted toward the electrophilic Pb center.^55,56^ As a result of the dimer formation, the overall complex becomes polar and the TEL moiety turns into a chiral structure, due to the asymmetric arrangements of the four ethyl ligands. However, the conformational arrangements of the ethyl ligands in the TEL-TFO complexes give rise to an extremely flat potential energy landscape. Within an energy window of just 1.0 kJ/mol, the CREST/GFN2-xTB and B3LYP-D4/def2-QZVP calculations revealed 75 structurally distinct isomers. Details of their spectroscopic parameters are provided in the Supporting Information. The B3LYP-D4/def2-QZVP calculations were also conducted with the utilization of the segmented all-electron relativistically contracted SARC-DKH-TZVPP basis set for the Pb atom, for use with the second-order Douglas-Kroll-Hess approach (DKH2),^57^ and the results were consistent.

The microwave spectrum is primarily characterized by one TEL-TFO isomer exhibiting solely *b*-type transitions arising from the electric dipole-moment component along the principal *b* axis (μ_*b*_) and following the Δ*K*_*a*_ = ± 1, Δ*K*_*c*_ = ± 1 selection rules. A section of the spectrum is displayed in Figure 2, with the zoomed-in view highlighting two rotational transitions originating from three naturally abundant Pb isotopologues, namely ^206^Pb (25.1%), ^207^Pb (22.1%), and ^208^Pb (52.4%). The observed transition frequencies are fitted for the three Pb isotopologues, respectively, with Watson’s *S*-reduced Hamiltonian in its *I* ^*r*^ representation implemented in Pickett’s SPFIT program.^58–60^ The spectroscopic parameters, including rotational constants (*A*, *B*, and *C*) and quartic centrifugal distortion constants (*D*_*J*_, *D*_*JK*_, *D*_*K*_, *d*_1_, and *d*_2_), are well determined and summarized in Table 1. Next, the atomic position of Pb in the principal inertial axis system is estimated using the Kraitchman equations, based on the rotational constants of the ^208^Pb and ^207^Pb isotopologues, assuming that the isotopic substitution at Pb does not alter the dimer structure.^60,61^ Kraitchman’s method provides only the absolute values of the coordinates, therefore, the signs were assigned based on the calculated equilibrium structure of the dimer.

**Figure 2:**
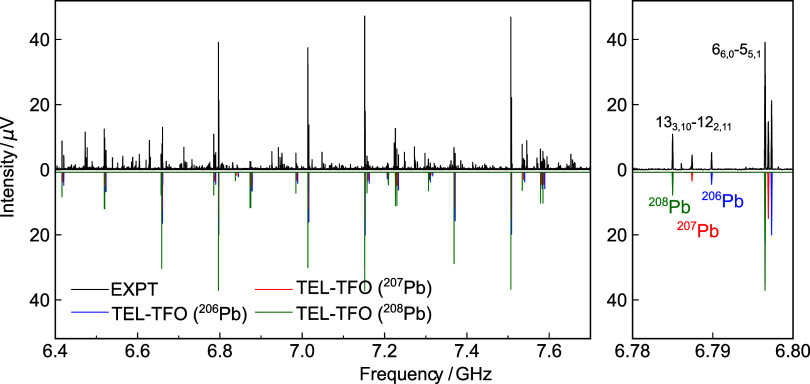
Portion of the microwave spectrum measured with the vapor mixture of tetraethyllead (TEL) and 2-(trifluoromethyl)­oxirane (TFO) in the frequency range of 2–8 GHz. The frequency-domain spectrum, obtained from the fast Fourier transformation of an average of 4.2 × 10^6^ FID acquisitions, is shown in the upper trace (EXPT). The transitions corresponding solely to TFO have been subtracted. The rotational spectra of the three Pb isotopologues (^206^Pb, ^207^Pb, and ^208^Pb) of the TEL-TFO dimer are provided in the lower trace for comparison, which are simulated based on the experimentally determined parameters at a rotational temperature of 1 K. A zoomed-in window is provided on the right side to highlight the *J*_*K*_*a*_′*K*_*c*_′_^′^ – *J*_*K*_*a*_″*K*_*c*_″_^″^ = 13_3,10_–12_2,11_ and 6_6,0_–5_5,1_ rotational transitions. The relative spectral strengths of the three isotopologues are simulated with their natural abundances (25.1%: 22.1%: 52.4%), showing good agreement with the experimental observations.

**Table 1:** Experimental Spectroscopic Parameters of the Three Pb Isotopologues of the TEL-TFO Dimer (^206^Pb, ^207^Pb, and ^208^Pb) Determined from SPFIT Least-Squares Fits, in Comparison with the Theoretical Values at the B3LYP-D4/def2-QZVP Level of Theory

	calc.	expt.		
parameters^a^	TEL-TFO-6 (^208^Pb)	TEL-TFO (^206^Pb)	TEL-TFO (^207^Pb)	TEL-TFO (^208^Pb)
*A*/MHz	588.1	601.779199(95)	601.75135(10)	601.723246(85)
*B*/MHz	181.2	182.027068(45)	181.918991(44)	181.811635(35)
*C*/MHz	173.4	173.562628(40)	173.466394(42)	173.370778(37)
*D*_*J*_/Hz		17.463(71)	17.523(68)	17.591(53)
*D*_*JK*_/Hz		4.59(31)	5.69(34)	4.27(21)
*D*_*K*_/Hz		70.3(12)	70.6(11)	67.5(10)
*d*_1_/Hz		–0.808(30)	–0.854(26)	–0.818(18)
*d*_2_/Hz		–0.074(16)	–0.032(10)	–0.0458(51)
N^b^		243	241	304
RMS^c^/kHz		4.7	5.0	4.8

a Theoretical rotational constants (*A*_e_, *B*_e_, and *C*_e_) are obtained from the equilibrium geometry (*r*_e_), while those determined from the experiment (*A*_0_, *B*_0_, and *C*_0_) are in the ground vibrational state (*r*_0_).

b Total number (N) of transition frequencies in the fit, which are all *b*-type rotational transitions.

c Root-mean-square deviation of the fit, RMS = $\sqrt{\frac{\sum {\left(\nu_{\mathrm{obs}}-\nu_{\mathrm{calc}}\right)}^{2}}{N}}$ .

To determine the corresponding overall geometry, the theoretical results for the 75 isomer candidates are systematically compared with experimentally derived values, including the rotational constants, planar moments, and the Pb position. The rotational constants describe the moments of inertia (*I*_*a*_, *I*_*b*_, and *I*_*c*_) in the dimension of frequency, and the planar moments (*P*_*aa*_, *P*_*bb*_, and *P*_*cc*_) quantify the mass distribution along the principal inertia axes.^62^ Deviations between theory and experiment, along with the applied equations, are provided in Supporting Table S1 and Figure S1. Among these evaluations, the Pb position, combined with the electric dipole-moment components, clearly establishes isomer-6 (also denoted herein as TEL-TFO-6) as the best match (see Figure 3a). This isomer also appears as a strong candidate when comparing rotational constants and planar moments, as shown in the Supporting Figure S1. The experimentally derived Pb position and the equilibrium structure of TEL-TFO-6 are depicted in Figure 3b, highlighting the evident agreement in the Pb atom position. The theoretical rotational constants of TEL-TFO-6 are also provided in Table 1 for comparison. Additionally, the predicted electric dipole-moment component |μ_*b*_| = 2.2 D is significantly larger than |μ_*a*_| = 0.0 D and |μ_*c*_| = 0.2 D, which agrees with the experimentally observed *b*-type rotational spectrum.

**Figure 3:**
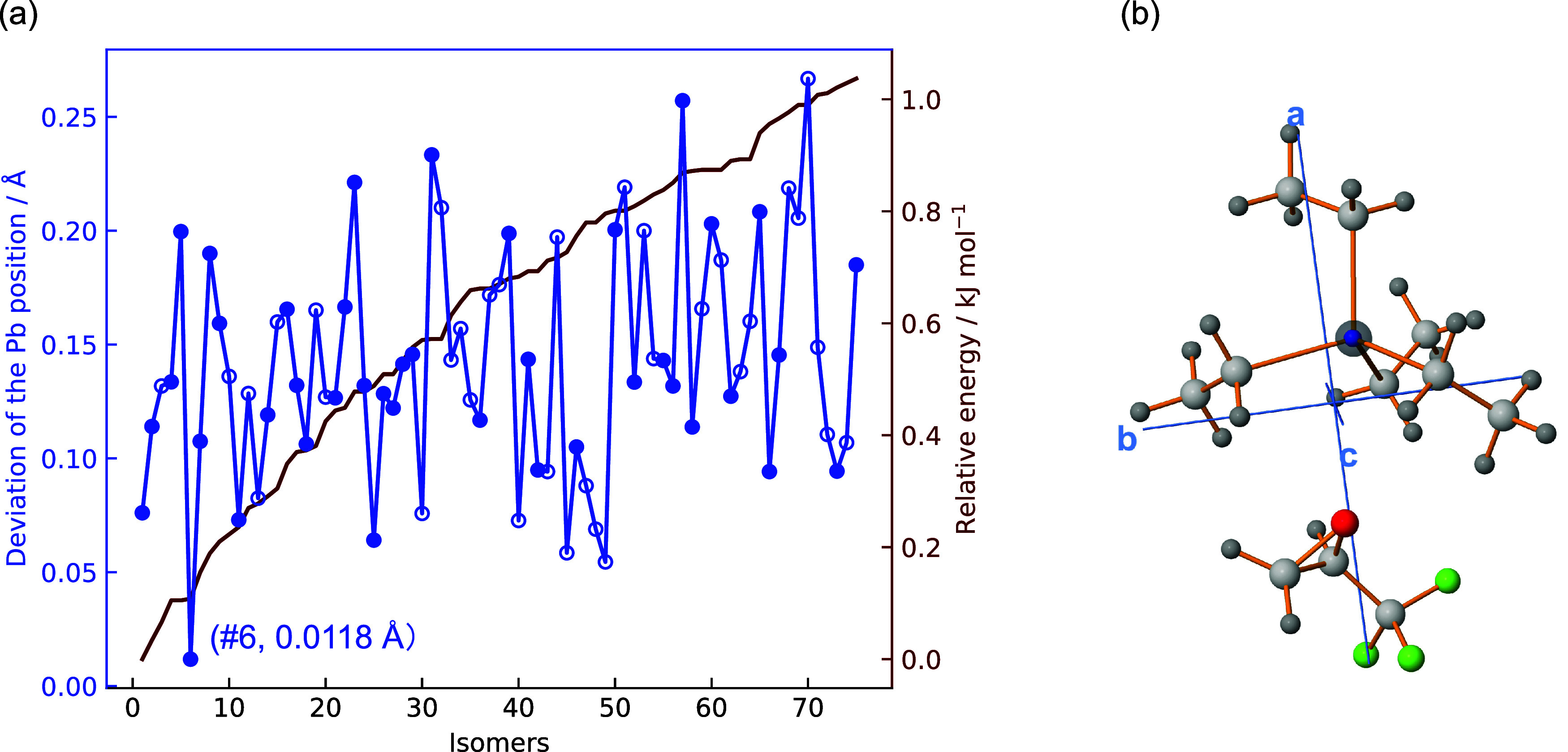
(a) Comparison of the differences between experimentally determined and theoretically predicted Pb positions for TEL-TFO isomers within 1.0 kJ/mol, computed at the B3LYP-D4/def2-QZVP level of theory with zero-point vibrational energy corrections estimated from the harmonic force constants. The solid-circle markers denote the isomers, whose μ_*b*_ dipole-moment components are more prominent than μ_*a*_ and μ_*c*_. (b) Equilibrium structure of the TEL-TFO-6 dimer in the principal axis system, where the Pb atom is displayed semitransparently and overlapped with the experimentally determined Pb position, represented by a solid sphere in blue.

In this structural arrangement, the dimer can potentially be stabilized through what has been referred to as a tetrel bond formed between the Pb and O atoms, accompanied by secondary hydrogen bonds between the O atom and adjacent C–H bonds (see Figure S2). According to the noncovalent interaction analysis, both types of interactions appear to be comparable in strength. The total interaction energy, including basis set superposition error (BSSE) corrections,^63^ is 18.8 kJ/mol, as computed at the B3LYP-D4/def2-QZVP level of theory. The Pb···O distance is 3.4 Å, close to the sum of van der Waals radii of Pb and O (∑*R*_vdW_ = 3.54 Å). The bond angle of O···Pb–C is nearly linear (176°), with C being the one situated along the extension of the O···Pb distance vector. Whereas this arrangement reflects the characteristic (quasi-)­linear nature ascribed to tetrel bonds, the relatively long internuclear distance indicates a weak tetrel bond strength. This behavior can be attributed to the limited electron-withdrawing ability of the ethyl groups, making TEL a weak tetrel-bond donor in the context of tetrel bonding, similar to the previously reported tetramethyllead (TML), Pb­(CH_3_)_4_.^56^

An ESP analysis, performed on the van der Waals surface of the TEL moiety in the dimer framework, reveals that all σ-holes around the Pb­(IV) center exhibit values below +62.5 kJ/mol, comparable to Pb­(CH_3_)_4_ (+54.4 kJ/mol) but substantially lower than those associated with stronger electron-withdrawing substituents, such as Pb­(CH_3_)_3_F (+184 kJ/mol).^56^ Of the four σ-holes present on TEL, the one closest to TFO in the dimer complex corresponds to the most positive site (+58.6 kJ/mol), whereas the remaining three show lower ESP values of 37.7, 51.0, and 54.0 kJ/mol, respectively. Future investigations could examine halogen-substituted TEL or TML derivatives, which can be expected to feature stronger interactions.

After excluding the transitions corresponding to TEL-TFO-6, a new species was identified in the residual spectrum and determined to be a neon complex of the TEL-TFO dimer. As shown in Figure 4, this trimer gives rise to two sets of Pb isotopologue patterns with an intensity ratio of approximately 4:1, attributed to the ^20^Ne and ^22^Ne isotopologues, respectively. Although the natural abundance ratio of ^20^Ne: ^22^Ne is about 9.8:1, the formation of weakly bound complexes enhances the prevalence of the heavier ^22^Ne isotopologue due to its slightly lower zero-point vibrational energy and, consequently, higher dissociation energy. The greater stabilization leads to an increased abundance of the ^22^Ne isotopologue in a supersonic expansion, compared to the natural abundance in the unbound form, which has been previously reported in other Ne-containing complexes.^64,65^

**Figure 4:**
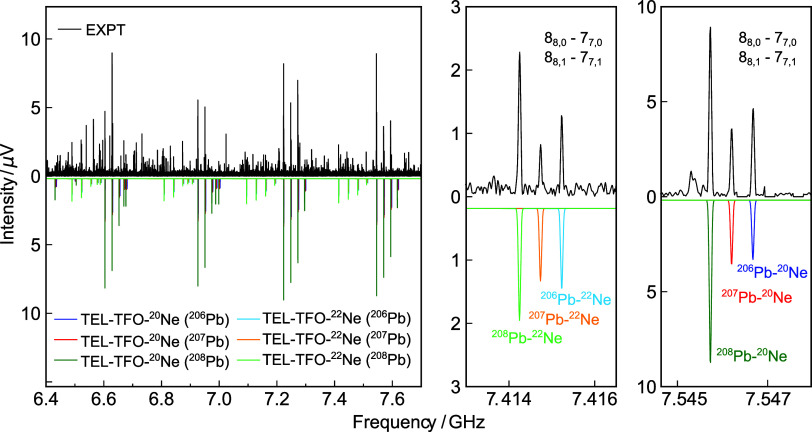
Spectral portion after the removal of the transitions assigned to the TEL-TFO dimer, illustrated in the same region as Figure 2. In the spectrum, the TEL-TFO-Ne trimers formed with ^20^Ne and ^22^Ne, respectively, were identified (simulation shown in the lower spectral part). Two zoomed-in windows are provided on the right side to highlight the degenerate 8_8,0_–7_7,0_ and 8_8,1_–7_7,1_ rotational transitions arising from the six doubly substituted isotopologues (^206/207/208^Pb–^22^Ne and ^206/207/208^Pb-^20^Ne).

The microwave spectra of the six doubly substituted Pb–Ne isotopologues of the TEL-TFO-Ne trimer are fitted individually, and the resulting spectroscopic parameters are given in Supporting Table S3. From the fitted rotational constants, the atomic positions of Pb and Ne are determined. However, because the Kraitchman equations only yield absolute values for the *r*_s_ coordinates, three possible geometries are found through constrained CREST searches, in which the TEL-TFO moiety was fixed to the TEL-TFO-6 dimer structure, as described in the theoretical details. These three candidates are isoenergetic at the B3LYP-D4/def2-QZVP level of theory. Notably, none of the fully relaxed CREST-ORCA calculations produce suitable isomer candidates. A comparison of the three candidates with the experimentally fitted results is provided in Table 2 for the ^208^Pb-^20^Ne isotopologues of the TEL-TFO-Ne trimer. Figure 5 presents the experimentally derived positions of the Pb and Ne atoms in comparison to the candidate isomers, with Ne situated in different quadrants around the TEL-TFO-6 dimer. Among them, isomer III matches most closely, however, isomers I and II remain viable due to the flexible nature of the Ne complexes. While the Ne position cannot be definitively determined, the observation of the TEL-TFO-Ne trimer still strongly supports the proposed structural assignment of the observed TEL-TFO dimer.

**Table 2:** Experimentally Determined Parameters of the ^208^Pb-^20^Ne Isotopologue of the TEL-TFO-Ne Trimer, in Comparison with the Theoretical Isomer Candidates Calculated at the B3LYP-D4/def2-QZVP Level of Theory

	I	II	III	expt.
Δ*E*_0_/kJ mol^–1^	0.0	0.0	0.2	
*A*/MHz	456.7	471.1	456.6	470.84754(19)
*B*/MHz	163.6	163.5	165.9	164.24345(10))
*C*/MHz	157.0	157.5	157.9	157.87779(10)
|μ_*a*_|, |μ_*b*_|, |μ_*c*_|/D	0, 0.8, 2.1	0, 1.3, 1.9	0, 0.7, 2.1	
N^a^				100
RMS^b^/kHz				6.0

Ne position^c^				
*a*/Å	–3.084	3.177	–3.008	±3.037(1)
*b*/Å	–3.264	–2.957	–3.216	±3.091(1)
*c*/Å	–0.377	–0.751	0.670	±0.650(6)

Pb position^d^				
*a*/Å	1.416	1.128	1.364	±1.379(1)
*b*/Å	0.190	0.121	–0.178	±0.174(13)
*c*/Å	0.109	–0.083	0.019	±0.022(104)

a Total number (*N*) of transition frequencies in the fit, which are all c-type rotational transitions.

b Root-mean-square deviation of the least-squares fit.

c Experimental *r*_*s*_ coordinates of Ne are derived in their absolute values, using the rotational constants of the ^208^Pb-^20^Ne and ^208^Pb–^22^Ne isotopologues.

d Experimental *r*_*s*_ coordinates of Pb are derived in their absolute values, using the ^207^Pb-^20^Ne and ^208^Pb-^20^Ne isotopologues.

**Figure 5:**
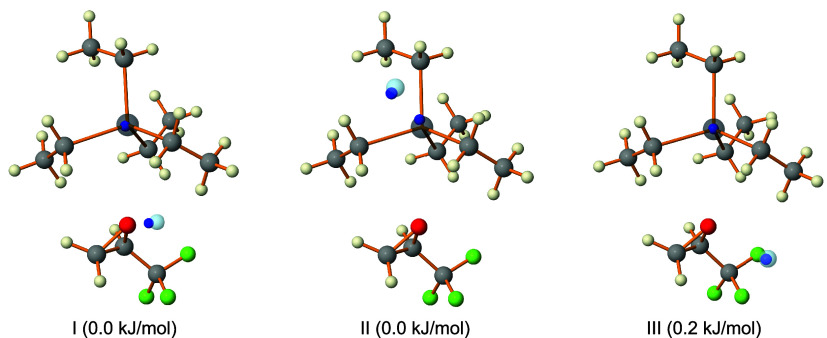
Equilibrium structures of the isomer candidates of the TEL-TFO-Ne trimer in the principal axis system, where the Pb and Ne atoms were displayed semitransparently and overlapped with the experimentally determined positions, represented by solid spheres in blue. Relative energies were computed at the B3LYP-D4/def2-QZVP level of theory with the inclusion of zero-point vibrational energy corrections.

After assignments of the TEL-TFO dimer and TEL-TFO-Ne trimer, approximately 600 lines with signal-to-noise ratios greater than 2 remain unassigned in the spectrum, which may arise from other dimer or trimer complexes. However, only 15 triplets are identified that could plausibly correspond to the characteristic pattern expected for the three Pb isotopologues, and their isotopic shifts are inconsistent. No further reliable assignments could therefore be made. The intensities of these remaining lines are one order of magnitude weaker than those of the assigned TEL-TFO dimer transitions, suggesting that other TEL-TFO isomers largely relax to TEL-TFO-6 during the supersonic expansion.

### Parity-Violating Effects

Using the experimentally observed structure of the TEL-TFO dimer, the PV energy shifts are calculated for the dimer as well as for its TEL and TFO subunits at various levels of theory. The results are summarized in Table 3, with a typical energy ordering observed as LDA > B3LYP ≈ MP2 > HF or reverse (see e.g., ref. 66). As expected, the TFO monomer exhibits only weak PV effects due to the absence of heavy atoms, whereas isolated TEL is achiral and therefore has an expectation value of zero for the parity-violating potential. Upon complex formation, however, the TEL unit becomes distorted from its achiral equilibrium structure, leading to nonzero PV energy shifts, *E*_pv_, for the TEL moiety taken from the complex as well as for the total complex.

**Table 3:** Calculated Total Parity-Violating Energy Shifts, *E*_pv_ (in *E*_h_), for the TEL-TFO Dimer (Results Are Individual Shifts for the Dimer with R-TFO, i.e., the Structure Shown in Figure 3b)

	HF	B3LYP	MP2	LDA
TEL-TFO dimer	2.940 × 10^–17^	4.396 × 10^–17^	4.971 × 10^–17^	5.497 × 10^–17^
TEL part	2.749 × 10^–17^	3.441 × 10^–17^	3.751 × 10^–17^	4.211 × 10^–17^
TFO part	–1.044 × 10^–20^	–1.482 × 10^–20^	–1.348 × 10^–20^	–1.699 × 10^–20^
*E*_int_	0.192 × 10^–17^	0.956 × 10^–17^	1.221 × 10^–17^	1.288 × 10^–17^

According to calculations, the TFO monomer yields an *E*_pv_ on the order of 1 × 10^–20^ *E*_h_, placing it between deuterated oxirane (*E*_pv_ = 9 × 10^–22^ *E*_h_) and fluorooxirane (7 × 10^–19^ *E*_h_).^67–69^ By forming the weakly bound dimer between TFO and TEL, the PV effects are increased by three orders of magnitude from the values of the monomer. This enhancement is primarily attributed to the distorted structure of TEL. By breaking the molecular symmetry via the intermolecular interactions with the chiral TFO tag, the TEL subunit becomes chiral as well. Since it contains a heavy Pb atom, the PV effect is strongly increased. Additionally, we give the energy difference *E*_int_ between *E*_pv_ of the dimer and the monomers

$$E_{\mathrm{int}}=E_{\mathrm{pv}}\left(\mathrm{dimer}\right)-E_{\mathrm{pv}}\left(\mathrm{TFO}\right)-E_{\mathrm{pv}}\left(\mathrm{TEL}\right)$$

in Table 3 (the monomer specification refers to the distorted monomer structures found in the dimer complex). *E*_int_ shows that even though the TFO monomer is relatively far away from the heavy center, is only bound via intermolecular interaction forces, and has comparatively negligible *E*_pv_ on its own, its effect is nevertheless an ≈ 25% increase of the PV energy shift at the MP2 level compared to the distorted TEL monomer. This enhancement (*E*_int_) by itself exceeds the *E*_pv_ of the TFO tag by three orders of magnitude.

The effect is comparable at the B3LYP and LDA levels of theory, whereas it is approximately four times weaker at the HF level. The difference between *E*_int_/*E*_pv_(dimer) at the HF and MP2 levels implies that the increase in *E*_pv_ can be attributed to electron correlation via intermolecular interaction forces.^70^ Further analysis beyond the scope of our present work, for instance within the framework of an energy decomposition analysis, could be attempted to disentangle intermolecular dispersion contributions from correlation effects arising from induced electrostatic and orbital relaxation terms.

Compared to specifically constructed chiral Pb compounds, such as PbHBrClF (*E*_pv_ = 1.582 × 10^–16^ *E*_h_),^13^ the PV energy shift in the TEL-TFO dimer is smaller. This is expected, as Pb is the only nonfirst-row element in the TEL-TFO complex and is spatially remote from the stereogenic center in TFO. Nevertheless, *E*_pv_(dimer) already surpasses the effects predicted in several other model molecules, as detailed in ref. 71. According to the single-center theorem,^4^ the PV energy shift is reduced in molecules containing a single main-group heavy center like TEL-TFO. This inhibition could be overcome and PV effects enhanced by incorporating a heavy-atom-containing complexing partner, such as readily available iodine compounds.^4,24,72^ Moreover, the detection of TEL-TFO-Ne complexes in this study suggests that mixing in heavy rare gases like Kr or Xe as part of the carrier gas could promote the formation of trimer complexes with a third heavy nucleus, potentially resulting in further enhancements.

## Conclusions

In summary, we investigated Pb-containing complexes, formed from tetraethyllead (TEL) and 2-(trifluoromethyl)­oxirane (TFO) in a Ne-seeded supersonic jet, using broadband chirped-pulse Fourier transform microwave (CP-FTMW) spectroscopy. Quantum-chemical calculations, employing the CREST/xTB and B3LYP-D4/def2-QZVP levels of theory, revealed a total of 75 structural isomers of the TEL-TFO dimer within an energy range of 1.0 kJ/mol, posing a significant challenge for spectroscopic interpretation. Through considerable effort, the true global-minimum configuration was identified in the rotational spectrum, characterized by its three monosubstituted ^206/207/208^Pb isotopologues in their natural abundance. Additionally, complexes of this TEL-TFO dimer with Ne were observed, featuring six doubly substituted ^206/207/208^Pb-^20/22^Ne isotopologues. The spectroscopic data allowed for the unambiguous determination of the TEL-TFO dimer structure, making it a study of great interest from both experimental and theoretical perspectives. Furthermore, parity-violating (PV) effects were calculated for this chiral dimer, with the chirality of the complex governed by the chiral binding partner, TFO. The induced parity violating potentials in the noncovalently bound dimeric complex exceed those of TFO by three orders of magnitude. These findings provide valuable insights into the emergence of PV effects in weakly bound complexes containing heavy atoms.

## Supplementary Material



## Notes

Detailed theoretical results (Table S1 and Figures S1–S3), Cartesian coordinates (Tables S2 and Tables S4–S6), experimentally determined spectroscopic parameters (Table S3), and experimental transition frequencies (Tables S7–S15) for the assigned TEL-TFO dimer and TEL-TFO-Ne trimer (PDF)
