# Supplementary material for: Chirality Induced in Tetraethyllead through Noncovalent Interactions with a Chiral Tag
Source: J Am Chem Soc. 2026 Feb 6;148(6):6551–9. doi: 10.1021/jacs.5c21116 (PMC12921877; doi:10.1021/jacs.5c21116)
Supplement: Supplementary file 1 [file ja5c21116_si_001.pdf]

Chirality Induced in Tetraethyllead through Non-covalent Interactions with a Chiral Tag

Wenhao Sun,[†] Steffen M. Giesen,[‡] Robert Berger,[*,‡] and Melanie Schnell[*,†,¶]

†*Deutsches Elektronen-Synchrotron DESY, Notkestr. 85, 22607 Hamburg, Germany*

‡*Fachbereich Chemie, Theoretische Chemie, Philipps-Universität Marburg, Hans-Meerwein-Str. 4, 35032 Marburg, Germany*

¶*Institute of Physical Chemistry, Christian-Albrechts-Universität zu Kiel, Max-Eyth-Str. 1, 24118 Kiel, Germany*

E-mail: robert.berger@uni-marburg.de; melanie.schnell@desy.de

Contents

1 TEL-TFO complexes. 3

1.1 Comparison between experimental and calculated results. 3

1.2 Experimentally assigned TEL-TFO dimer. 8

1.3 Characterization of non-covalent interactions. 10

2 TEL-TFO-Ne complexes. 12

2.1 Doubly substituted isotopologues of the TEL-TFO-Ne trimer. 12

2.2 Candidates for the experimentally assigned TEL-TFO-Ne trimer. 12

3 Measured rotational transitions. 18

3.1 Frequency lists of TEL-TFO complexes. 18

3.2 Frequency lists of TEL-TFO-Ne complexes. 56

1 TEL-TFO complexes.

1.1 Comparison between experimental and calculated results.

Table S1: Theoretical spectroscopic and structural properties of the TEL-TFO isomers, within an energy window of 1.0 kJ/mol, calculated at the B3LYP-D4/def2-QZVP level of theory.

#	ΔE_0[1]	ΔE_e[2]	A_e[3]	B_e[3]	C_e[3]	$\mid \mu_a \mid$[4]	$\mid \mu_b \mid$[4]	$\mid \mu_c \mid$[4]	ΔRC[5]	ΔPM[6]	ΔX_{Pb}[7]
1	0.0	0.3	566.3	189.2	171.3	0.0	1.6	1.3	0.073	0.240	0.116
2	0.0	0.1	572.2	200.5	190.2	1.4	2.4	0.4	0.150	0.138	0.186
3	0.1	0.0	607.1	190.2	184.0	1.2	1.1	2.0	0.077	0.097	0.170
4	0.1	0.5	580.1	186.2	173.7	0.0	2.2	0.2	0.043	0.103	0.194
5	0.1	0.5	587.3	202.1	180.0	1.6	2.4	0.4	0.120	0.302	0.284
6	0.1	0.2	588.1	181.2	173.4	0.0	2.2	0.2	0.023	0.043	0.015
7	0.2	0.2	558.8	185.9	177.0	0.2	1.9	0.7	0.078	0.118	0.181
8	0.2	0.4	592.9	198.4	176.4	1.3	2.4	0.2	0.094	0.321	0.282
9	0.2	0.9	577.1	185.5	171.0	0.1	1.8	1.0	0.048	0.161	0.225
10	0.2	0.4	616.7	181.8	178.8	0.1	1.5	1.4	0.040	0.150	0.221
11	0.2	0.0	632.0	177.2	165.9	0.1	2.1	0.2	0.071	0.150	0.097
12	0.3	0.6	625.0	180.5	169.5	0.1	1.0	2.0	0.045	0.114	0.176
13	0.3	0.5	614.3	179.1	168.5	0.3	0.6	1.8	0.038	0.092	0.116
14	0.3	0.1	563.4	189.3	174.8	0.1	1.8	1.3	0.076	0.158	0.163
15	0.3	0.2	610.1	194.2	183.5	1.5	1.4	1.8	0.091	0.078	0.182
16	0.3	0.9	571.6	189.1	173.4	0.1	1.8	1.1	0.064	0.181	0.232
17	0.4	0.4	579.2	185.5	177.2	0.0	1.8	1.3	0.048	0.070	0.143
18	0.4	0.5	609.6	187.5	176.1	1.0	2.2	0.7	0.037	0.080	0.129
19	0.4	0.2	607.9	195.5	184.8	1.5	1.3	1.8	0.100	0.083	0.187

#	ΔE_0[1]	ΔE_e[2]	A_e[3]	B_e[3]	C_e[3]	$\mid \mu_a \mid$[4]	$\mid \mu_b \mid$[4]	$\mid \mu_c \mid$[4]	ΔRC[5]	ΔPM[6]	ΔX_{Pb}[7]
20	0.4	0.7	621.7	180.2	169.0	0.2	0.9	1.9	0.043	0.116	0.177
21	0.4	0.2	553.9	186.6	173.9	0.3	1.8	1.2	0.084	0.142	0.153
22	0.4	0.4	523.6	201.8	198.2	1.5	2.3	0.5	0.222	0.345	0.257
23	0.5	0.9	593.4	191.5	183.1	1.2	2.4	0.2	0.079	0.074	0.366
24	0.5	0.6	577.4	194.9	188.1	1.1	2.2	0.8	0.118	0.144	0.200
25	0.5	0.5	604.7	180.9	165.5	0.1	2.0	0.9	0.046	0.225	0.072
26	0.5	0.8	586.0	184.1	179.4	0.0	2.0	0.9	0.045	0.133	0.174
27	0.5	0.3	562.6	192.9	176.7	0.1	1.8	1.2	0.091	0.187	0.181
28	0.5	0.5	591.2	188.8	171.7	0.1	1.8	1.3	0.044	0.226	0.204
29	0.6	0.8	593.1	198.5	183.0	1.2	2.3	0.3	0.108	0.159	0.187
30	0.6	0.7	605.5	166.0	156.2	0.3	0.7	2.1	0.132	0.159	0.110
31	0.6	0.4	618.9	180.3	171.3	0.3	2.0	0.2	0.032	0.055	0.391
32	0.6	0.8	611.4	175.1	170.0	0.2	1.4	1.4	0.045	0.089	0.341
33	0.6	1.0	571.7	181.1	171.9	0.3	1.0	1.8	0.051	0.073	0.230
34	0.6	0.6	597.6	198.4	181.7	1.5	2.0	1.0	0.103	0.188	0.174
35	0.7	1.2	582.0	179.7	173.7	0.3	1.5	1.6	0.035	0.094	0.194
36	0.7	0.7	640.2	171.2	168.9	0.0	1.9	1.1	0.090	0.172	0.190
37	0.7	1.3	581.3	180.6	178.8	0.0	0.7	2.2	0.047	0.212	0.243
38	0.7	0.8	556.9	199.7	193.1	1.4	1.5	1.7	0.168	0.209	0.272
39	0.7	0.8	597.8	176.5	169.7	0.0	1.9	1.2	0.037	0.048	0.263
40	0.7	0.5	578.9	185.9	179.9	0.1	1.0	1.9	0.058	0.118	0.082
41	0.7	0.8	591.5	200.3	184.0	1.3	2.4	0.3	0.120	0.175	0.181
42	0.7	1.1	599.0	185.0	180.2	0.7	2.2	0.8	0.044	0.118	0.127
43	0.7	0.7	572.6	185.5	177.9	0.1	1.1	1.8	0.059	0.099	0.109
44	0.7	0.5	608.6	183.9	178.2	0.3	1.4	1.5	0.032	0.081	0.294
45	0.7	0.9	567.4	180.2	172.8	0.0	0.7	2.0	0.058	0.100	0.098

#	ΔE_0[1]	ΔE_e[2]	A_e[3]	B_e[3]	C_e[3]	$\mid \mu_a \mid$[4]	$\mid \mu_b \mid$[4]	$\mid \mu_c \mid$[4]	ΔRC[5]	ΔPM[6]	ΔX_{Pb}[7]
46	0.8	0.5	574.3	188.7	171.1	0.0	2.0	1.1	0.061	0.235	0.174
47	0.8	0.9	578.4	185.6	178.7	0.0	1.1	1.9	0.054	0.097	0.097
48	0.8	0.8	568.5	185.7	170.4	0.2	0.9	1.8	0.062	0.184	0.112
49	0.8	0.7	569.4	183.9	169.9	0.3	0.8	1.8	0.059	0.156	0.080
50	0.8	1.1	595.2	199.0	176.3	1.5	2.3	0.4	0.097	0.336	0.316
51	0.8	1.0	576.5	180.6	174.5	0.2	0.5	2.1	0.043	0.104	0.330
52	0.8	1.2	579.5	182.1	176.2	0.3	1.9	1.2	0.041	0.109	0.211
53	0.8	1.2	564.3	179.4	173.9	0.1	1.5	1.7	0.064	0.146	0.303
54	0.8	1.4	607.5	173.9	166.4	0.3	1.3	1.6	0.060	0.054	0.222
55	0.8	1.1	565.2	193.0	185.2	1.0	1.9	1.3	0.110	0.143	0.216
56	0.9	1.1	573.7	200.4	190.5	1.6	2.3	0.3	0.150	0.138	0.215
57	0.9	1.2	601.1	192.5	184.0	1.5	2.3	0.4	0.085	0.071	0.434
58	0.9	0.5	570.1	192.9	173.9	0.0	2.0	1.2	0.081	0.255	0.193
59	0.9	0.7	573.5	198.2	191.6	1.5	1.9	1.3	0.146	0.169	0.233
60	0.9	1.4	561.1	201.9	186.0	1.4	2.4	0.2	0.149	0.182	0.328
61	0.9	1.1	594.3	170.9	162.2	0.4	0.7	2.0	0.089	0.086	0.290
62	0.9	1.4	531.9	188.4	173.0	0.1	2.0	0.8	0.122	0.222	0.198
63	0.9	1.2	626.5	185.4	173.9	0.8	0.0	2.4	0.046	0.111	0.212
64	0.9	1.2	598.9	179.6	172.5	0.0	1.1	2.1	0.014	0.039	0.263
65	0.9	1.2	596.1	200.6	178.2	1.5	2.3	0.5	0.108	0.323	0.330
66	1.0	1.3	515.7	185.3	171.9	0.2	1.8	1.0	0.145	0.243	0.156
67	1.0	1.0	580.5	185.0	172.3	0.2	2.4	0.2	0.040	0.111	0.210
68	1.0	1.0	585.2	193.1	192.0	1.2	1.5	1.8	0.127	0.246	0.371
69	1.0	0.9	609.2	179.7	167.8	0.2	0.7	1.8	0.036	0.123	0.343
70	1.0	0.7	595.0	195.3	192.0	1.3	1.9	1.3	0.131	0.193	0.462
71	1.0	1.2	618.9	166.1	154.9	0.6	0.4	2.0	0.140	0.211	0.219

#	ΔE_0[1]	ΔE_e[2]	A_e[3]	B_e[3]	C_e[3]	$\mid \mu_a \mid$[4]	$\mid \mu_b \mid$[4]	$\mid \mu_c \mid$[4]	ΔRC[5]	ΔPM[6]	ΔX_Pb[7]
72	1.0	1.1	644.1	183.3	172.1	1.2	0.6	2.3	0.071	0.143	0.161
73	1.0	1.0	573.5	184.8	169.3	0.2	2.1	0.8	0.055	0.193	0.162
74	1.0	1.5	616.6	174.4	164.8	0.6	0.6	1.8	0.068	0.097	0.131
75	1.0	1.1	590.6	196.4	179.3	1.2	2.3	0.0	0.089	0.198	0.306

[1] Relative energies in units of kJ mol^{-1}, with vibrational zero-point energies accounted for.

[2] Relative electronic energies in units of kJ mol^{-1}.

[3] Rotational constants of the equilibrium structure of the ^{208}Pb isotopologue, in units of MHz.

[4] Electric dipole-moment components along the principal a-, b- and c-axis, respectively, in units of D.

[5] Weighted deviations of the theoretical rotational constants from the experimental determined values of the ^{208}Pb isotopologue. $\Delta RC = \sqrt{\sum_N (\frac{N_e - N_0}{N_0})^2}, N \in \{A, B, C\}$, where $A_0 = 601.723246(85)$ MHz, $B_0 = 181.811635(35)$ MHz, and $C_0 = 173.370778(37)$ MHz.

[6] Weighted deviations of the theoretical planar moments from the experimental determined values of the ^{208}Pb isotopologue. $\Delta PM = \sqrt{\sum_N (\frac{N_e - N_0}{N_0})^2}, N \in \{P_{aa}, P_{bb}, P_{cc}\}$, where P^0_{aa} = 2427.40844(42) amu·Å^2, P^0_{bb} = 487.60995(42) amu·Å^2, P^0_{cc} = 352.27618(42) amu·Å^2. Planar moments, also known as second moments, are calculated by $P_{xx} = \sum m_i x_i^2$ (x = a, b, c), which describe the mass distribution along the principal a, b-, and c-axis, respectively.

[7] Deviations of the theoretical Pb atom position from the experimental determined values of the ^{208}Pb isotopologue, in units of Å. $\Delta X_\mathrm{Pb} = \sqrt{\sum_N (|N_e| - |N_0|)^2}, N \in \{a_\mathrm{Pb}, b_\mathrm{Pb}, c_\mathrm{Pb}\}$, where $a^0_\mathrm{Pb} = 1.27050$ Å, $b^0_\mathrm{Pb} = -0.04905$ Å, $c^0_\mathrm{Pb} = 0.19110$ Å. The experimental position of the Pb atom is determined by the Kraitchman equations using the two sets of rotational constants of the ^{207}Pb and ^{208}Pb isotopologues.

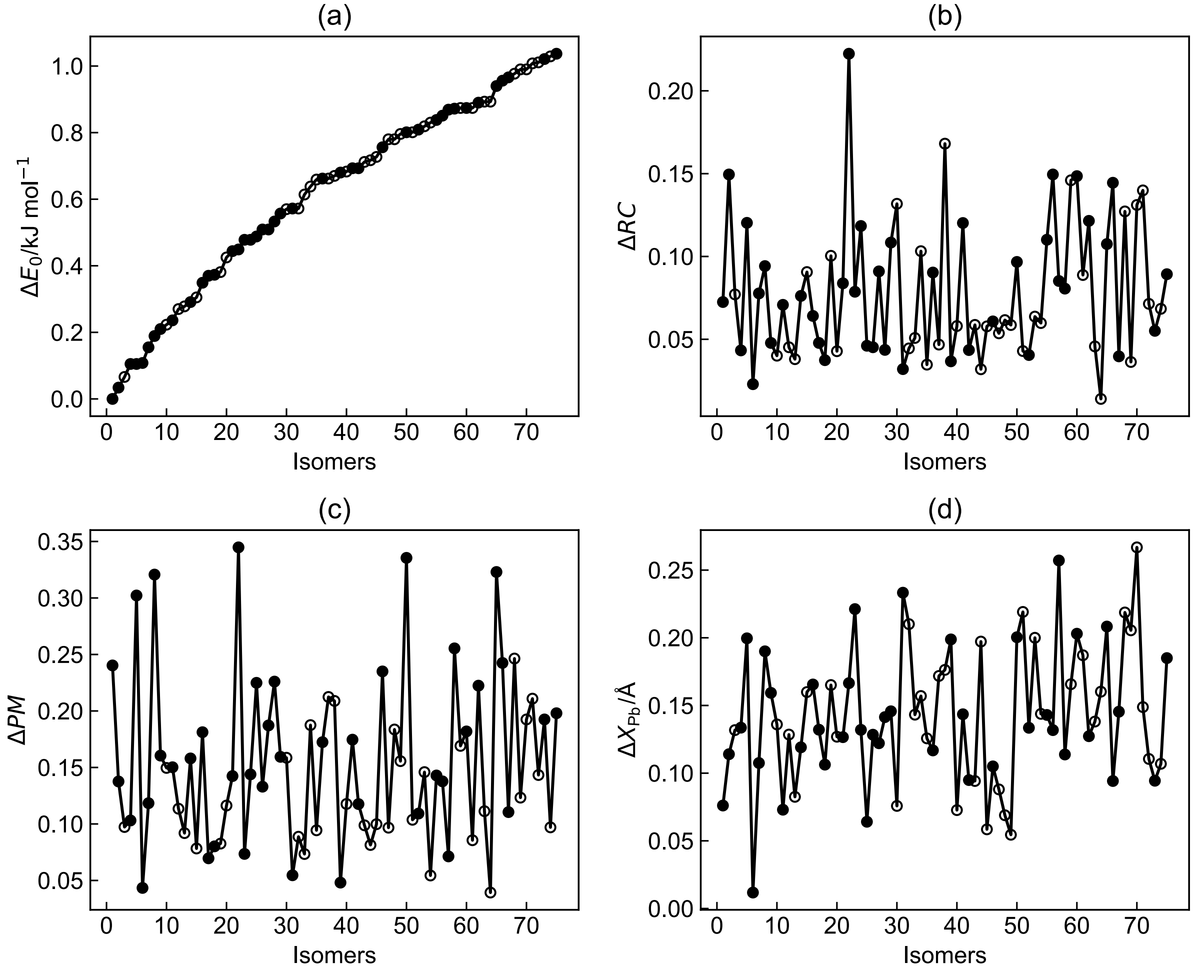

Figure S1: Relative energies of the TEL-TFO isomers within 1.0 kJ/mol (a), and the deviations of their theoretical molecular properties from the corresponding experimental results, including rotational constants (b), planar moments (c), and the Pb positions (d). The solid-circle markers denote the isomers, whose μ_b dipole-moment components are more prominent than μ_a and μ_c.

1.2 Experimentally assigned TEL-TFO dimer.

Table S2: Cartesian coordinates (in Å) of the experimentally assigned TEL-TFO dimer (isomer-6 in Table S1), which is used in the PV calculations.

Atoms	X	Y	Z
Pb	1.261320	-0.064153	0.190609
C	3.468259	-0.373011	-0.288974
C	4.007189	0.686695	-1.244436
H	3.997106	-0.358679	0.663412
H	3.566038	-1.374873	-0.704877
H	3.896552	1.692298	-0.835130
H	3.484798	0.670272	-2.202506
H	5.070591	0.541744	-1.456241
C	0.103816	-0.610589	-1.692064
C	0.852834	-1.603414	-2.574257
H	-0.847223	-1.018250	-1.356877
H	-0.095940	0.319481	-2.223221
H	1.049166	-2.539546	-2.049694
H	1.816167	-1.210211	-2.900963
H	0.283421	-1.852737	-3.474225
C	0.996986	2.148095	0.658777
C	0.546622	2.967145	-0.544958
H	0.278616	2.198275	1.475065
H	1.954895	2.500385	1.039229
H	1.283003	2.939804	-1.349175
H	0.390186	4.019573	-0.288928
H	-0.388912	2.594874	-0.966134

Atoms	X	Y	Z
C	0.599420	-1.390566	1.909682
C	-0.349116	-2.497778	1.466331
H	1.507867	-1.790260	2.357919
H	0.128055	-0.727319	2.632440
H	-1.256816	-2.091921	1.021623
H	-0.654100	-3.122343	2.310852
H	0.111701	-3.157235	0.729278
C	-2.886625	0.840822	-0.219183
C	-2.778315	1.781035	0.893326
O	-2.009900	0.571927	0.857061
H	-2.419612	1.058440	-1.172903
H	-2.208925	2.690043	0.747660
H	-3.551788	1.806749	1.649491
C	-4.068880	-0.087745	-0.347385
F	-4.683553	-0.310846	0.822934
F	-4.973298	0.436561	-1.198772
F	-3.692381	-1.281187	-0.842658

1.3 Characterization of non-covalent interactions.

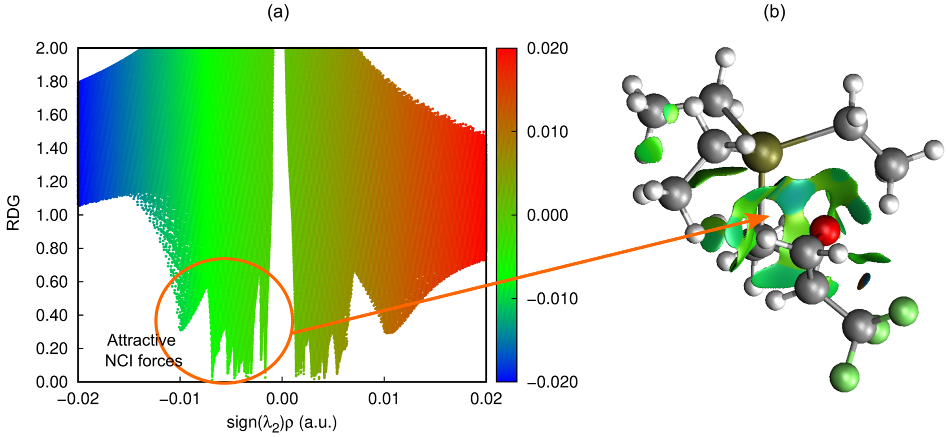

Figure S2: (a) Scatter map between reduced density gradient (RDG) and sign$(\lambda_2)\rho$, where λ_2 is the second-largest eigenvalue of the electron density Hessian matrix, sign(λ_2) represents the sign of λ_2, and ρ denotes the electron density, showing non-covalent interactions in the TEL-TFO dimer. The color bar ranges from -0.02 to +0.02 E_h. A detailed description of the analysis can be found in Ref. 1. (b) NCI plot showing the interactions between TEL and TFO, with isosurfaces of RDG = 0.4 colored by sign$(\lambda_2)\rho$ according to the color bar in panel (a).

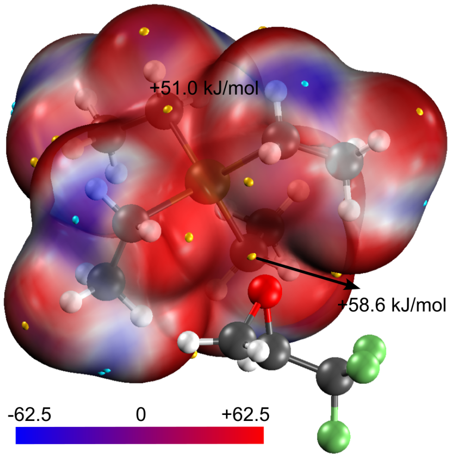

Figure S3: Molecular electrostatic potential (ESP) on the van der Waals surface of the TEL moiety in the TEL-TFO dimer. The energy ranges from -62.5 to +62.5 kJ/mol and is represented by a color scale ranging from blue to red. The yellow and cyan dots shown on the ESP correspond to the local maxima and minima, respectively.

(1) Tian Lu and Qinxue Chen. Visualization analysis of weak interactions in chemical systems. In Manuel Yáñez and Russell J. Boyd, editors, *Comprehensive Computational Chemistry*, pp 240–264. Elsevier, Oxford, First Edition, 2024. ISBN 978-0-12-823256-9. DOI: 10.1016/B978-0-12-821978-2.00076-3.

2 TEL-TFO-Ne complexes.

2.1 Doubly substituted isotopologues of the TEL-TFO-Ne trimer.

Table S3: Experimental spectroscopic parameters of the six doubly substituted isotopologues of the TEL-TFO-Ne trimer (^{206}Pb/^{207}Pb/^{208}Pb-^{20}Ne/^{22}Ne) determined from SPFIT least-squares fits, using Watson's S-reduced Hamiltonian in its I^r representation.

Parameters	^{206}Pb-^{20}Ne	^{206}Pb-^{22}Ne	^{207}Pb-^{20}Ne	^{207}Pb-^{22}Ne	^{208}Pb-^{20}Ne	^{208}Pb-^{22}Ne
A_0/MHz	470.87441(27)	462.39764(30)	470.86096(48)	462.38598(45)	470.84754(19)	462.37322(33)
B_0/MHz	164.44780(17)	163.42863(50)	164.34495(25)	163.32531(39)	164.24345(10)	163.22237(40)
C_0/MHz	158.07026(16)	156.24073(39)	157.97337(27)	156.14464(36)	157.87779(10)	156.04966(28)
D_J/kHz	0.01501(40)	0.0168(12)	0.01368(62)	0.01520(86)	0.01487(19)	0.01436(89)
D_{JK}/kHz	0.0337(18)	0.0318(33)	0.0368(23)	0.0403(25)	0.03269(79)	0.0430(28)
D_K/kHz	0.1062(29)	0.1064(38)	0.1035(58)	0.1107(45)	0.1054(17)	0.1089(39)
N[a]	73	41	73	39	100	50
RMS[b]/kHz	5.3	5.1	6.8	6.1	6.0	6.4

[a] Total number (N) of lines in the fit, which are all c-type rotational transitions.

[b] Root-mean-square deviation of the fit, RMS = $\sqrt{\frac{\sum(\nu_{obs}-\nu_{calc})^2}{N}}$.

2.2 Candidates for the experimentally assigned TEL-TFO-Ne trimer.

Table S4: Cartesian coordinates (in Å) of candidate-I of the experimentally assigned TEL-TFO-Ne trimer.

Atoms	X	Y	Z
Pb	1.415560	0.189593	0.109117
C	3.558606	-0.568813	0.278233
C	4.010315	-1.313624	-0.973927
H	4.181867	0.303764	0.471062
H	3.601446	-1.205385	1.161023
H	3.955166	-0.680938	-1.861461
H	3.392337	-2.192369	-1.166315
H	5.044515	-1.660431	-0.890840

Atoms	X	Y	Z
C	0.066231	-1.640999	0.225334
C	0.709437	-2.798119	0.981820
H	-0.850575	-1.313473	0.710201
H	-0.176572	-1.923373	-0.798532
H	0.946862	-2.523678	2.010671
H	1.639304	-3.123095	0.513717
H	0.047968	-3.668144	1.025791
C	1.228370	1.176067	-1.934040
C	0.667637	0.245799	-3.003102
H	0.597017	2.050437	-1.786752
H	2.224275	1.527525	-2.200780
H	1.315550	-0.615993	-3.168779
H	0.554682	0.751920	-3.966831
H	-0.312169	-0.149046	-2.728516
C	0.912040	1.620584	1.798052
C	-0.096137	1.038286	2.781248
H	1.854770	1.868129	2.283921
H	0.530387	2.520889	1.320219
H	-1.038716	0.796714	2.291699
H	-0.321442	1.744340	3.585701
H	0.273999	0.125110	3.250043
C	-2.745248	0.425022	-0.821986
C	-2.510185	1.707914	-1.480414
O	-1.763080	1.311604	-0.323735
H	-2.379087	-0.495728	-1.261105
H	-1.952945	1.716851	-2.408450

Atoms	X	Y	Z
H	-3.199273	2.526210	-1.318322
C	-3.944175	0.211725	0.068797
F	-4.436051	1.358316	0.559899
F	-4.927000	-0.399334	-0.622945
F	-3.633842	-0.577705	1.113415
Ne	-3.083709	-3.263811	-0.377139

Table S5: Cartesian coordinates (in Å) of candidate-II of the experimentally assigned TEL-TFO-Ne trimer.

Atoms	X	Y	Z
Pb	1.125976	0.120430	-0.082130
C	3.269888	0.894020	-0.091959
C	3.750149	1.286786	1.301302
H	3.883103	0.100447	-0.517427
H	3.300559	1.738104	-0.779615
H	3.705658	0.445759	1.995380
H	3.143015	2.087500	1.727207
H	4.785511	1.639899	1.291159
C	-0.217407	1.923715	0.279878
C	0.422860	3.231361	-0.173078
H	-1.140744	1.727256	-0.260408
H	-0.446276	1.942394	1.344930
H	0.648232	3.220541	-1.240467
H	1.359247	3.427983	0.350345

Atoms	X	Y	Z
H	-0.234680	4.086548	0.007728
C	0.965594	-1.337767	1.659193
C	0.420819	-0.696843	2.930217
H	0.331503	-2.151331	1.311503
H	1.964668	-1.741134	1.818430
H	1.073368	0.099174	3.291196
H	0.318730	-1.422251	3.743268
H	-0.561534	-0.248202	2.772595
C	0.592370	-0.850520	-2.063289
C	-0.428388	-0.045687	-2.858280
H	1.527089	-0.971515	-2.608742
H	0.214137	-1.840372	-1.815179
H	-1.363283	0.065627	-2.310592
H	-0.666368	-0.532256	-3.808541
H	-0.063642	0.955622	-3.092705
C	-3.021530	-0.339067	0.818591
C	-2.783303	-1.743511	1.139773
O	-2.048158	-1.076419	0.106055
H	-2.645973	0.445898	1.464968
H	-2.211621	-1.979911	2.028075
H	-3.476347	-2.497915	0.791492
C	-4.230232	0.088207	0.023031
F	-4.731294	-0.900156	-0.731237
F	-5.202857	0.508688	0.856554
F	-3.929386	1.112586	-0.796211
Ne	3.177651	-2.957268	-0.751140

Table S6: Cartesian coordinates (in Å) of candidate-III of the experimentally assigned TEL-TFO-Ne trimer.

Atoms	X	Y	Z
Pb	1.364250	-0.178565	0.019479
C	3.598595	-0.128349	-0.429601
C	4.271755	1.125350	0.119406
H	4.022350	-1.032736	0.005753
H	3.707525	-0.201494	-1.510830
H	4.150164	1.207702	1.200798
H	3.855592	2.033114	-0.320905
H	5.346532	1.133320	-0.084318
C	0.395295	1.378922	-1.330868
C	1.217818	1.664534	-2.582603
H	-0.590006	0.994472	-1.584984
H	0.262072	2.278176	-0.730214
H	1.348562	0.768498	-3.191084
H	2.214321	2.034513	-2.338740
H	0.739568	2.416506	-3.216763
C	1.122972	0.429146	2.200598
C	0.815495	1.911059	2.379494
H	0.329926	-0.200254	2.599975
H	2.049256	0.155269	2.703984
H	1.619752	2.539760	1.995116
H	0.676358	2.176058	3.432167
H	-0.092074	2.206781	1.850297

Atoms	X	Y	Z
C	0.487079	-2.228550	-0.406318
C	-0.445358	-2.232673	-1.611291
H	1.332443	-2.900611	-0.546759
H	-0.033414	-2.523790	0.502523
H	-1.295876	-1.568602	-1.462791
H	-0.846705	-3.232507	-1.799986
H	0.064812	-1.914392	-2.521784
C	-2.674596	1.006992	0.574741
C	-2.672416	0.436648	1.919659
O	-1.928977	-0.162322	0.851152
H	-2.099341	1.897674	0.349596
H	-2.072847	0.914201	2.684160
H	-3.525711	-0.136260	2.258097
C	-3.854078	0.825770	-0.348242
F	-4.595584	-0.247003	-0.037686
F	-4.650796	1.911765	-0.293565
F	-3.450281	0.685093	-1.624472
Ne	-3.008218	-3.215824	0.670013

3 Measured rotational transitions.

3.1 Frequency lists of TEL-TFO complexes.

Table S7: Assigned rotational transitions for the ^{206}Pb isotopologue of isomer TEL-TFO-6.

J'	$K_a{}'$	$K_c{}'$	J''	$K_a{}''$	$K_c{}''$	ν_{obs}/MHz	ν_{calc}/MHz	$\Delta\nu$/kHz
13	3	10	13	2	11	2015.087	2015.090	-3.9
12	3	9	12	2	10	2040.142	2040.147	-4.4
11	3	8	11	2	9	2061.130	2061.133	-3.3
10	3	7	10	2	8	2078.147	2078.148	-1.3
9	3	6	9	2	7	2091.458	2091.461	-3.6
8	3	5	8	2	6	2101.474	2101.472	1.6
7	3	4	7	2	5	2108.665	2108.664	1.3
9	1	8	8	2	7	2111.919	2111.918	0.9
6	3	3	6	2	4	2113.556	2113.553	2.4
6	3	4	6	2	5	2122.282	2122.282	-0.9
7	3	5	7	2	6	2124.247	2124.247	-0.2
8	3	6	8	2	7	2127.167	2127.168	-1.8
9	3	7	9	2	8	2131.293	2131.294	-0.5
5	1	5	4	0	4	2139.471	2139.470	1.3
7	0	7	6	1	6	2140.013	2140.013	-0.0
12	3	10	12	2	11	2153.592	2153.589	3.2
13	3	11	13	2	12	2165.270	2165.269	0.5
14	3	12	14	2	13	2179.548	2179.550	-1.5
15	3	13	15	2	14	2196.710	2196.710	0.7
16	3	14	16	2	15	2217.016	2217.016	-0.6

J'	K_a'	K_c'	J''	K_a''	K_c''	ν_{obs}/MHz	ν_{calc}/MHz	$\Delta\nu$/kHz
12	2	10	11	3	9	2236.366	2236.361	5.6
11	1	11	10	2	8	2288.420	2288.426	-5.4
3	2	2	2	1	1	2326.030	2326.022	7.7
20	3	18	20	2	19	2334.480	2334.482	-2.6
3	2	1	2	1	2	2352.050	2352.049	1.1
13	2	12	12	3	9	2455.781	2455.783	-1.2
6	1	6	5	0	5	2471.208	2471.205	3.1
10	1	9	9	2	8	2506.539	2506.539	-0.6
8	0	8	7	1	7	2516.392	2516.391	0.8
12	1	12	11	2	9	2563.924	2563.926	-2.2
13	2	11	12	3	10	2621.172	2621.166	6.7
4	2	3	3	1	2	2668.892	2668.894	-1.8
4	2	2	3	1	3	2721.583	2721.578	4.5
14	2	13	13	3	10	2794.317	2794.314	3.0
7	1	7	6	0	6	2799.977	2799.976	0.8
20	4	16	20	3	17	2860.247	2860.248	-1.5
19	4	15	19	3	16	2882.899	2882.901	-1.6
9	0	9	8	1	8	2893.522	2893.521	1.1
18	4	14	18	3	15	2901.711	2901.711	0.5
11	1	10	10	2	9	2904.279	2904.281	-2.0
17	4	13	17	3	14	2917.093	2917.091	2.5
16	4	12	16	3	13	2929.478	2929.476	1.6
15	4	11	15	3	12	2939.296	2939.299	-4.0
14	4	10	14	3	11	2946.970	2946.971	-0.2
13	4	9	13	3	10	2952.867	2952.866	1.4
12	4	8	12	3	9	2957.319	2957.319	-0.0

J'	$K_a{}'$	$K_c{}'$	J''	$K_a{}''$	$K_c{}''$	ν_{obs}/MHz	ν_{calc}/MHz	$\Delta\nu$/kHz
17	4	14	17	3	15	2959.048	2959.045	2.3
16	4	13	16	3	14	2959.311	2959.313	-1.9
18	4	15	18	3	16	2959.377	2959.374	3.3
15	4	12	15	3	13	2959.993	2959.991	1.7
11	4	7	11	3	8	2960.623	2960.622	1.5
14	4	11	14	3	12	2960.927	2960.925	2.1
9	4	5	9	3	6	2964.720	2964.721	-1.9
10	4	7	10	3	8	2965.012	2965.016	-4.1
8	4	4	8	3	5	2965.893	2965.894	-0.6
8	4	5	8	3	6	2966.430	2966.436	-6.3
5	2	4	4	1	3	3007.526	3007.526	-0.2
14	2	12	13	3	11	3011.481	3011.487	-6.9
17	3	15	16	4	12	3091.469	3091.470	-1.6
5	2	3	4	1	4	3096.588	3096.586	1.7
8	1	8	7	0	7	3126.312	3126.315	-2.5
15	2	14	14	3	11	3128.425	3128.423	1.3
17	3	14	16	4	13	3136.413	3136.413	-0.1
3	3	1	2	2	0	3186.636	3186.636	-0.1
3	3	0	2	2	1	3186.764	3186.764	0.2
10	0	10	9	1	9	3270.706	3270.718	-12.5
12	1	11	11	2	10	3304.867	3304.869	-2.1
6	2	5	5	1	4	3341.944	3341.945	-1.2
15	2	13	14	3	12	3407.456	3407.452	3.8
9	1	9	8	0	8	3450.824	3450.823	1.7
6	2	4	5	1	5	3477.707	3477.706	1.3
4	3	2	3	2	1	3541.957	3541.966	-9.1

J'	$K_a{}'$	$K_c{}'$	J''	$K_a{}''$	$K_c{}''$	ν_{obs}/MHz	ν_{calc}/MHz	$\Delta\nu$/kHz
4	3	1	3	2	2	3542.615	3542.608	7.5
11	0	11	10	1	10	3647.327	3647.327	0.9
7	2	6	6	1	5	3672.196	3672.196	0.6
13	1	12	12	2	11	3707.984	3707.981	3.2
10	1	10	9	0	9	3774.148	3774.148	0.0
22	5	17	22	4	18	3774.354	3774.357	-3.7
25	5	21	25	4	22	3774.778	3774.790	-12.3
17	2	16	16	3	13	3780.221	3780.223	-2.0
23	5	19	23	4	20	3783.432	3783.430	2.4
22	5	18	22	4	19	3787.581	3787.578	3.0
20	5	15	20	4	16	3788.790	3788.788	2.8
21	5	17	21	4	18	3791.509	3791.502	6.7
19	5	14	19	4	15	3794.202	3794.196	5.4
20	5	16	20	4	17	3795.142	3795.147	-4.2
19	5	15	19	4	16	3798.475	3798.474	1.6
18	5	13	18	4	14	3798.652	3798.651	1.0
17	5	12	17	4	13	3802.304	3802.303	0.8
17	5	13	17	4	14	3804.102	3804.105	-2.6
16	5	11	16	4	12	3805.282	3805.282	0.1
16	5	12	16	4	13	3806.410	3806.404	6.0
15	5	10	15	4	11	3807.701	3807.697	4.1
15	5	11	15	4	12	3808.379	3808.374	4.9
14	5	10	14	4	11	3810.029	3810.033	-4.2
13	5	8	13	4	9	3811.198	3811.187	10.7
8	5	4	8	4	5	3815.003	3815.005	-1.9
8	5	3	8	4	4	3815.003	3815.001	2.6

J'	$K_a{}'$	$K_c{}'$	J''	$K_a{}''$	$K_c{}''$	ν_{obs}/MHz	ν_{calc}/MHz	$\Delta\nu$/kHz
7	5	3	7	4	4	3815.275	3815.275	-0.0
7	5	2	7	4	3	3815.275	3815.273	1.5
5	5	1	5	4	2	3815.562	3815.571	-8.1
7	2	5	6	1	6	3865.680	3865.679	1.1
19	3	16	18	4	15	3886.641	3886.645	-4.3
5	3	3	4	2	2	3896.804	3896.807	-3.5
5	3	2	4	2	3	3898.739	3898.740	-0.2
8	2	7	7	1	6	3998.340	3998.340	-0.2
12	0	12	11	1	11	4022.762	4022.758	4.2
11	1	11	10	0	10	4096.952	4096.954	-2.4
6	3	4	5	2	3	4250.808	4250.810	-2.2
6	3	3	5	2	4	4255.335	4255.335	0.1
8	2	6	7	1	7	4261.335	4261.333	1.8
9	2	8	8	1	7	4320.470	4320.467	3.1
4	4	1	3	3	0	4390.252	4390.250	2.2
4	4	0	3	3	1	4390.252	4390.251	1.0
13	0	13	12	1	12	4396.518	4396.521	-3.0
12	1	12	11	0	11	4419.878	4419.883	-4.8
15	1	14	14	2	13	4520.219	4520.209	10.8
7	3	5	6	2	4	4603.535	4603.537	-1.8
7	3	4	6	2	5	4612.617	4612.614	3.1
10	2	9	9	1	8	4638.685	4638.690	-4.9
15	6	9	15	5	10	4659.609	4659.599	10.5
15	6	10	15	5	11	4659.609	4659.610	-1.3
14	6	9	14	5	10	4660.543	4660.545	-2.7
14	6	8	14	5	9	4660.543	4660.540	3.1

J'	$K_a{}'$	$K_c{}'$	J''	$K_a{}''$	$K_c{}''$	ν_{obs}/MHz	ν_{calc}/MHz	$\Delta\nu$/kHz
12	6	6	12	5	7	4661.916	4661.914	1.6
12	6	7	12	5	8	4661.916	4661.915	0.4
11	6	5	11	5	6	4662.393	4662.394	-1.3
11	6	6	11	5	7	4662.393	4662.395	-1.9
10	6	5	10	5	6	4662.768	4662.764	4.0
10	6	4	10	5	5	4662.768	4662.764	4.2
9	6	3	9	5	4	4663.039	4663.042	-2.9
9	6	4	9	5	5	4663.039	4663.042	-2.9
8	6	2	8	5	3	4663.241	4663.244	-3.0
8	6	3	8	5	4	4663.241	4663.244	-3.0
7	6	2	7	5	3	4663.382	4663.387	-4.2
7	6	1	7	5	2	4663.382	4663.387	-4.2
6	6	1	6	5	2	4663.475	4663.482	-7.3
6	6	0	6	5	1	4663.475	4663.482	-7.3
9	2	7	8	1	8	4665.555	4665.555	-0.2
13	1	13	12	0	12	4743.518	4743.517	0.9
5	4	2	4	3	1	4745.868	4745.860	8.4
5	4	1	4	3	2	4745.868	4745.868	0.1
14	0	14	13	1	13	4768.244	4768.248	-3.6
16	1	15	15	2	14	4928.391	4928.393	-2.2
11	2	10	10	1	9	4953.161	4953.161	-0.0
8	3	6	7	2	5	4954.471	4954.470	0.4
8	3	5	7	2	6	4970.849	4970.849	0.2
14	1	14	13	0	13	5068.349	5068.350	-1.0
10	2	8	9	1	9	5079.243	5079.247	-4.1
6	4	2	5	3	3	5101.442	5101.459	-16.9

J'	K_a'	K_c'	J''	K_a''	K_c''	ν_{obs}/MHz	ν_{calc}/MHz	$\Delta\nu$/kHz
15	0	15	14	1	14	5137.700	5137.698	2.0
12	2	11	11	1	10	5264.065	5264.068	-2.5
9	3	7	8	2	6	5303.030	5303.029	0.8
9	3	6	8	2	7	5330.363	5330.363	-0.1
17	1	16	16	2	15	5337.244	5337.240	3.7
15	1	15	14	0	14	5394.756	5394.760	-3.6
7	4	4	6	3	3	5456.913	5456.905	7.0
7	4	3	6	3	4	5457.007	5457.007	0.2
21	7	15	21	6	16	5502.738	5502.742	-4.2
21	7	14	21	6	15	5502.738	5502.735	3.0
11	2	9	10	1	10	5503.276	5503.284	-7.5
20	7	13	20	6	14	5504.261	5504.261	0.2
20	7	14	20	6	15	5504.261	5504.265	-3.8
19	7	12	19	6	13	5505.578	5505.578	-0.0
19	7	13	19	6	14	5505.578	5505.580	-2.2
18	7	12	18	6	13	5506.706	5506.707	-0.5
18	7	11	18	6	12	5506.706	5506.706	0.7
16	7	9	16	6	10	5508.475	5508.471	3.6
16	7	10	16	6	11	5508.475	5508.471	3.3
15	7	8	15	6	9	5509.146	5509.144	2.1
15	7	9	15	6	10	5509.146	5509.144	2.0
14	7	8	14	6	9	5509.700	5509.698	2.4
14	7	7	14	6	8	5509.700	5509.698	2.5
13	7	7	13	6	8	5510.150	5510.148	1.4
13	7	6	13	6	7	5510.150	5510.148	1.4
12	7	6	12	6	7	5510.511	5510.509	2.1

J'	K_a'	K_c'	J''	K_a''	K_c''	ν_{obs}/MHz	ν_{calc}/MHz	$\Delta\nu$/kHz
12	7	5	12	6	6	5510.511	5510.509	2.1
11	7	4	11	6	5	5510.794	5510.793	0.6
11	7	5	11	6	6	5510.794	5510.793	0.6
10	7	4	10	6	5	5511.012	5511.012	-0.3
10	7	3	10	6	4	5511.012	5511.012	-0.3
9	7	2	9	6	3	5511.181	5511.177	4.5
9	7	3	9	6	4	5511.181	5511.177	4.5
8	7	1	8	6	2	5511.299	5511.297	2.1
8	7	2	8	6	3	5511.299	5511.297	2.1
7	7	1	7	6	2	5511.383	5511.381	1.6
7	7	0	7	6	1	5511.383	5511.381	1.6
13	2	12	12	1	11	5571.628	5571.645	-17.2
5	5	1	4	4	0	5593.795	5593.791	4.0
5	5	0	4	4	1	5593.795	5593.791	4.0
10	3	8	9	2	7	5648.592	5648.593	-1.4
10	3	7	9	2	8	5691.544	5691.540	4.2
16	1	16	15	0	15	5723.012	5723.001	11.4
18	1	17	17	2	16	5746.149	5746.147	2.3
8	4	5	7	3	4	5812.229	5812.242	-14.0
8	4	4	7	3	5	5812.503	5812.496	7.4
17	0	17	16	1	16	5869.427	5869.428	-0.7
14	2	13	13	1	12	5876.176	5876.178	-2.9
6	5	2	5	4	1	5949.409	5949.409	-0.1
6	5	1	5	4	2	5949.409	5949.409	-0.2
11	3	9	10	2	8	5990.529	5990.533	-3.6
17	1	17	16	0	16	6053.206	6053.208	-1.9

J'	$K_a{}'$	$K_c{}'$	J''	$K_a{}''$	$K_c{}''$	$\nu_{\rm obs}$/MHz	$\nu_{\rm calc}$/MHz	$\Delta\nu$/kHz
11	3	8	10	2	9	6054.827	6054.824	2.4
19	1	18	18	2	17	6154.469	6154.474	-4.7
9	4	6	8	3	5	6167.349	6167.358	-9.3
9	4	5	8	3	6	6167.923	6167.916	6.7
15	2	14	14	1	13	6178.012	6178.012	0.6
18	0	18	17	1	17	6231.797	6231.788	8.8
7	5	2	6	4	3	6305.006	6305.008	-2.6
7	5	3	6	4	2	6305.006	6305.008	-2.1
12	3	10	11	2	9	6328.246	6328.246	0.1
23	8	15	23	7	16	6351.372	6351.373	-1.7
23	8	16	23	7	17	6351.372	6351.374	-2.1
22	8	15	22	7	16	6352.657	6352.657	0.3
22	8	14	22	7	15	6352.657	6352.656	0.5
21	8	13	21	7	14	6353.776	6353.779	-2.5
21	8	14	21	7	15	6353.776	6353.779	-2.6
20	8	13	20	7	14	6354.758	6354.754	3.9
20	8	12	20	7	13	6354.758	6354.754	3.9
19	8	11	19	7	12	6355.596	6355.597	-0.8
19	8	12	19	7	13	6355.596	6355.597	-0.8
18	8	11	18	7	12	6356.324	6356.319	4.3
18	8	10	18	7	11	6356.324	6356.319	4.3
17	8	9	17	7	10	6356.933	6356.934	-0.5
17	8	10	17	7	11	6356.933	6356.934	-0.5
16	8	8	16	7	9	6357.448	6357.451	-3.6
16	8	9	16	7	10	6357.448	6357.451	-3.6
15	8	7	15	7	8	6357.870	6357.883	-12.6

J'	$K_a{}'$	$K_c{}'$	J''	$K_a{}''$	$K_c{}''$	ν_{obs}/MHz	ν_{calc}/MHz	$\Delta\nu$/kHz
15	8	8	15	7	9	6357.870	6357.883	-12.6
14	8	6	14	7	7	6358.234	6358.239	-4.4
14	8	7	14	7	8	6358.234	6358.239	-4.4
12	8	4	12	7	5	6358.760	6358.760	-0.8
12	8	5	12	7	6	6358.760	6358.760	-0.8
10	8	3	10	7	4	6359.085	6359.084	1.2
10	8	2	10	7	3	6359.085	6359.084	1.2
8	8	0	8	7	1	6359.272	6359.268	4.2
8	8	1	8	7	2	6359.272	6359.268	4.2
18	1	18	17	0	17	6385.409	6385.409	-0.1
12	3	9	11	2	10	6420.734	6420.731	2.9
16	2	15	15	1	14	6477.549	6477.549	0.1
10	4	7	9	3	6	6522.141	6522.148	-6.7
10	4	6	9	3	7	6523.266	6523.267	-0.6
20	1	19	19	2	18	6561.570	6561.563	6.7
19	0	19	18	1	18	6591.988	6591.988	-0.6
8	5	4	7	4	3	6660.573	6660.573	-0.5
8	5	3	7	4	4	6660.573	6660.575	-2.0
13	3	11	12	2	10	6661.198	6661.199	-0.8
19	1	19	18	0	18	6719.549	6719.547	1.5
17	2	16	16	1	15	6775.261	6775.258	2.8
13	3	10	12	2	11	6789.847	6789.845	1.9
6	6	0	5	5	1	6797.324	6797.321	2.8
6	6	1	5	5	0	6797.324	6797.321	2.8
11	4	8	10	3	7	6876.495	6876.478	17.5
20	0	20	19	1	19	6950.216	6950.217	-1.5

J'	K_a'	K_c'	J''	K_a''	K_c''	ν_{obs}/MHz	ν_{calc}/MHz	$\Delta\nu$/kHz
21	1	20	20	2	19	6966.769	6966.765	4.0
14	3	12	13	2	11	6988.944	6988.955	-10.3
9	5	4	8	4	5	7016.089	7016.091	-2.4
9	5	5	8	4	4	7016.089	7016.087	2.2
20	1	20	19	0	19	7055.511	7055.505	6.2
18	2	17	17	1	16	7071.667	7071.667	0.1
7	6	1	6	5	2	7152.943	7152.939	4.6
7	6	2	6	5	1	7152.943	7152.939	4.6
14	3	11	13	2	12	7162.829	7162.828	1.4
12	4	9	11	3	8	7230.180	7230.178	1.5
12	4	8	11	3	9	7233.834	7233.827	7.7
21	0	21	20	1	20	7306.688	7306.683	4.3
15	3	13	14	2	12	7311.210	7311.204	6.0
15	2	13	14	1	14	7317.018	7317.021	-2.8
19	2	18	18	1	17	7367.370	7367.357	12.9
10	5	5	9	4	6	7371.536	7371.539	-3.4
10	5	6	9	4	5	7371.536	7371.527	8.6
21	1	21	20	0	20	7393.117	7393.123	-5.8
8	6	2	7	5	3	7508.543	7508.544	-0.4
8	6	3	7	5	2	7508.543	7508.544	-0.4
15	3	12	14	2	13	7540.412	7540.418	-5.8
13	4	10	12	3	9	7583.024	7583.035	-11.6
13	4	9	12	3	10	7589.119	7589.122	-2.9
16	3	14	15	2	13	7627.771	7627.773	-1.0
22	0	22	21	1	21	7661.595	7661.599	-3.3
20	2	19	19	1	18	7662.949	7662.955	-5.7

J'	$K_a{}'$	$K_c{}'$	J''	$K_a{}''$	$K_c{}''$	ν_{obs}/MHz	ν_{calc}/MHz	$\Delta\nu$/kHz
11	5	6	10	4	7	7726.882	7726.896	-14.5
11	5	7	10	4	6	7726.882	7726.868	13.6
22	1	22	21	0	21	7732.223	7732.226	-2.6
16	2	14	15	1	15	7802.109	7802.119	-9.9
9	6	3	8	5	4	7864.123	7864.128	-4.8
9	6	4	8	5	3	7864.123	7864.128	-4.8
16	3	13	15	2	14	7923.438	7923.428	9.6
14	4	11	13	3	10	7934.792	7934.789	2.8
17	3	15	16	2	14	7938.623	7938.621	1.9
14	4	10	13	3	11	7944.531	7944.529	1.7
21	2	20	20	1	19	7959.112	7959.108	4.4
7	7	0	6	6	1	8000.832	8000.838	-5.6
7	7	1	6	6	0	8000.832	8000.838	-5.6
23	0	23	22	1	22	8015.159	8015.170	-11.3
10	6	4	9	5	5	8219.697	8219.680	16.4

Table S8: Assigned rotational transitions for the ^{207}Pb isotopologue of isomer TEL-TFO-6.

J'	$K_a{}'$	$K_c{}'$	J''	$K_a{}''$	$K_c{}''$	ν_{obs}/MHz	ν_{calc}/MHz	$\Delta\nu$/kHz
13	3	10	13	2	11	2015.734	2015.729	4.7
10	3	7	10	2	8	2078.632	2078.636	-3.3
9	3	6	9	2	7	2091.915	2091.913	1.7
8	3	5	8	2	6	2101.896	2101.897	-0.7
7	3	4	7	2	5	2109.072	2109.068	4.8
9	1	8	8	2	7	2109.612	2109.611	1.3

J'	$K_a{}'$	$K_c{}'$	J''	$K_a{}''$	$K_c{}''$	$\nu_{\rm obs}$/MHz	$\nu_{\rm calc}$/MHz	$\Delta\nu$/kHz
12	2	11	11	3	8	2110.804	2110.802	1.9
6	3	3	6	2	4	2113.945	2113.943	1.4
5	3	2	5	2	3	2117.040	2117.038	2.1
5	3	3	5	2	4	2121.415	2121.417	-1.3
6	3	4	6	2	5	2122.645	2122.647	-1.6
7	3	5	7	2	6	2124.606	2124.606	0.7
8	3	6	8	2	7	2127.517	2127.519	-1.1
9	3	7	9	2	8	2131.628	2131.632	-3.9
10	3	8	10	2	9	2137.213	2137.209	3.6
7	0	7	6	1	6	2138.425	2138.425	-0.3
5	1	5	4	0	4	2138.608	2138.608	0.1
12	3	10	12	2	11	2153.863	2153.865	-1.4
13	3	11	13	2	12	2165.504	2165.513	-8.4
15	3	13	15	2	14	2196.869	2196.868	1.1
16	3	14	16	2	15	2217.121	2217.121	0.1
12	2	10	11	3	9	2233.294	2233.291	3.6
21	2	20	21	1	21	2280.356	2280.360	-3.3
3	2	2	2	1	1	2325.651	2325.650	0.5
20	3	18	20	2	19	2334.288	2334.290	-2.0
3	2	1	2	1	2	2351.637	2351.639	-2.0
21	3	19	21	2	20	2373.607	2373.618	-11.1
6	1	6	5	0	5	2470.173	2470.170	2.9
10	1	9	9	2	8	2503.983	2503.979	3.4
8	0	8	7	1	7	2514.585	2514.583	1.4
12	1	12	11	2	9	2562.003	2561.999	3.8
18	4	15	17	5	12	2599.821	2599.810	10.6

J'	K_a'	K_c'	J''	K_a''	K_c''	ν_{obs}/MHz	ν_{calc}/MHz	$\Delta\nu$/kHz
13	2	11	12	3	10	2617.814	2617.818	-4.2
4	2	3	3	1	2	2668.332	2668.335	-3.3
4	2	2	3	1	3	2720.943	2720.943	0.8
23	4	19	23	3	20	2767.364	2767.369	-5.2
14	2	13	13	3	10	2791.280	2791.278	2.1
7	1	7	6	0	6	2798.770	2798.770	-0.3
20	4	16	20	3	17	2861.142	2861.142	-0.4
19	4	15	19	3	16	2883.714	2883.718	-3.6
9	0	9	8	1	8	2891.496	2891.497	-0.2
11	1	10	10	2	9	2901.468	2901.465	3.1
18	4	14	18	3	15	2902.462	2902.463	-0.8
17	4	13	17	3	14	2917.802	2917.789	12.9
16	4	12	16	3	13	2930.133	2930.130	2.6
15	4	11	15	3	12	2939.915	2939.918	-3.2
14	4	10	14	3	11	2947.559	2947.562	-2.4
13	4	9	13	3	10	2953.439	2953.436	3.4
12	4	8	12	3	9	2957.876	2957.874	1.4
17	4	14	17	3	15	2959.565	2959.564	0.8
16	4	13	16	3	14	2959.835	2959.837	-2.7
15	4	12	15	3	13	2960.519	2960.520	-0.9
11	4	7	11	3	8	2961.168	2961.165	2.7
13	4	10	13	3	11	2962.514	2962.514	0.3
20	4	17	20	3	18	2963.160	2963.159	1.4
12	4	9	12	3	10	2963.597	2963.595	2.9
11	4	8	11	3	9	2964.621	2964.621	0.0
9	4	5	9	3	6	2965.260	2965.251	8.9

J'	K_a'	K_c'	J''	K_a''	K_c''	ν_{obs}/MHz	ν_{calc}/MHz	$\Delta\nu$/kHz
10	4	7	10	3	8	2965.541	2965.542	-0.6
9	4	6	9	3	7	2966.320	2966.326	-5.5
8	4	4	8	3	5	2966.428	2966.420	8.3
24	4	21	24	3	22	2987.435	2987.430	5.4
5	2	4	4	1	3	3006.787	3006.787	-0.2
14	2	12	13	3	11	3007.850	3007.852	-2.4
5	2	3	4	1	4	3095.717	3095.715	2.0
8	1	8	7	0	7	3124.934	3124.938	-3.7
15	2	14	14	3	11	3125.247	3125.249	-2.7
17	3	14	16	4	13	3132.210	3132.209	0.8
3	3	1	2	2	0	3186.396	3186.395	1.4
3	3	0	2	2	1	3186.516	3186.522	-5.9
10	0	10	9	1	9	3268.482	3268.482	0.0
12	1	11	11	2	10	3301.797	3301.797	0.7
6	2	5	5	1	4	3341.029	3341.031	-1.9
15	2	13	14	3	12	3403.524	3403.520	3.6
9	1	9	8	0	8	3449.279	3449.275	4.4
16	2	15	15	3	12	3454.049	3454.045	3.6
6	2	4	5	1	5	3476.591	3476.588	2.7
18	3	15	17	4	14	3505.054	3505.059	-5.2
4	3	2	3	2	1	3541.512	3541.521	-9.0
4	3	1	3	2	2	3542.169	3542.161	7.7
11	0	11	10	1	10	3644.884	3644.885	-1.9
7	2	6	6	1	5	3671.113	3671.112	0.2
13	1	12	12	2	11	3704.652	3704.652	-0.3
23	5	18	23	4	19	3765.721	3765.727	-5.8

J'	$K_a{}'$	$K_c{}'$	J''	$K_a{}''$	$K_c{}''$	ν_{obs}/MHz	ν_{calc}/MHz	$\Delta\nu$/kHz
10	1	10	9	0	9	3772.425	3772.426	-0.5
21	5	16	21	4	17	3783.032	3783.024	7.6
19	5	14	19	4	15	3794.932	3794.933	-0.5
20	5	16	20	4	17	3795.867	3795.865	2.2
19	3	17	18	4	14	3797.359	3797.361	-2.4
19	5	15	19	4	16	3799.191	3799.185	5.5
18	5	13	18	4	14	3799.378	3799.372	6.0
18	5	14	18	4	15	3802.165	3802.167	-2.0
16	5	11	16	4	12	3805.980	3805.981	-1.1
16	5	12	16	4	13	3807.106	3807.097	9.0
9	5	5	9	4	6	3815.278	3815.294	-16.6
9	5	4	9	4	5	3815.278	3815.282	-4.9
8	5	4	8	4	5	3815.673	3815.676	-2.6
8	5	3	8	4	4	3815.673	3815.671	1.9
6	5	2	6	4	3	3816.123	3816.126	-2.3
6	5	1	6	4	2	3816.123	3816.125	-1.9
5	5	0	5	4	1	3816.238	3816.240	-2.2
5	5	1	5	4	2	3816.238	3816.240	-2.3
7	2	5	6	1	6	3864.301	3864.300	0.3
5	3	3	4	2	2	3896.157	3896.160	-2.9
5	3	2	4	2	3	3898.086	3898.087	-1.2
8	2	7	7	1	6	3997.093	3997.093	-0.6
12	0	12	11	1	11	4020.121	4020.118	2.5
11	1	11	10	0	10	4095.048	4095.054	-6.0
6	3	4	5	2	3	4249.959	4249.964	-4.4
6	3	3	5	2	4	4254.473	4254.474	-1.6

J'	$K_a{}'$	$K_c{}'$	J''	$K_a{}''$	$K_c{}''$	ν_{obs}/MHz	ν_{calc}/MHz	$\Delta\nu$/kHz
8	2	6	7	1	7	4259.682	4259.679	2.7
9	2	8	8	1	7	4319.063	4319.061	1.7
4	4	1	3	3	0	4389.955	4389.953	2.1
4	4	0	3	3	1	4389.955	4389.954	0.9
13	0	13	12	1	12	4393.693	4393.690	2.6
12	1	12	11	0	11	4417.806	4417.799	6.6
15	1	14	14	2	13	4516.369	4516.373	-4.4
7	3	5	6	2	4	4602.492	4602.494	-2.8
7	3	4	6	2	5	4611.547	4611.544	3.2
10	2	9	9	1	8	4637.126	4637.131	-4.7
14	6	9	14	5	10	4661.377	4661.370	7.5
14	6	8	14	5	9	4661.377	4661.364	13.3
13	6	7	13	5	8	4662.119	4662.126	-6.2
13	6	8	13	5	9	4662.119	4662.128	-9.0
11	6	6	11	5	7	4663.217	4663.214	2.6
11	6	5	11	5	6	4663.217	4663.214	3.1
9	6	4	9	5	5	4663.859	4663.860	-1.0
9	6	3	9	5	4	4663.859	4663.860	-1.0
7	6	1	7	5	2	4664.201	4664.204	-3.2
7	6	2	7	5	3	4664.201	4664.204	-3.2
6	6	1	6	5	2	4664.295	4664.300	-5.0
6	6	0	6	5	1	4664.295	4664.300	-5.0
13	1	13	12	0	12	4741.246	4741.245	0.9
5	4	1	4	3	2	4745.361	4745.366	-4.8
5	4	2	4	3	1	4745.361	4745.358	3.6
14	0	14	13	1	13	4765.232	4765.231	1.1

J'	$K_a{}'$	$K_c{}'$	J''	$K_a{}''$	$K_c{}''$	ν_{obs}/MHz	ν_{calc}/MHz	$\Delta\nu$/kHz
16	1	15	15	2	14	4924.309	4924.310	-1.2
11	2	10	10	1	9	4951.458	4951.452	6.3
8	3	6	7	2	5	4953.238	4953.237	0.8
8	3	5	7	2	6	4969.560	4969.566	-6.1
14	1	14	13	0	13	5065.879	5065.883	-4.2
10	2	8	9	1	9	5076.985	5076.984	1.1
15	0	15	14	1	14	5134.503	5134.501	1.8
12	2	11	11	1	10	5262.217	5262.212	4.9
9	3	7	8	2	6	5301.614	5301.612	1.9
9	3	6	8	2	7	5328.863	5328.864	-1.2
17	1	16	16	2	15	5332.919	5332.917	1.8
15	1	15	14	0	14	5392.089	5392.092	-3.5
7	4	4	6	3	3	5456.009	5455.995	13.6
7	4	3	6	3	4	5456.094	5456.096	-2.1
11	2	9	10	1	10	5500.687	5500.686	0.6
16	0	16	15	1	15	5501.389	5501.387	2.0
22	7	16	22	6	17	5501.976	5501.980	-4.1
21	7	15	21	6	16	5503.725	5503.728	-2.2
21	7	14	21	6	15	5503.725	5503.721	4.9
20	7	14	20	6	15	5505.248	5505.247	0.8
20	7	13	20	6	14	5505.248	5505.243	4.8
19	7	13	19	6	14	5506.563	5506.558	4.8
19	7	12	19	6	13	5506.563	5506.556	7.0
16	7	10	16	6	11	5509.447	5509.443	4.3
16	7	9	16	6	10	5509.447	5509.442	4.6
14	7	8	14	6	9	5510.670	5510.666	3.2

J'	K_a'	K_c'	J''	K_a''	K_c''	ν_{obs}/MHz	ν_{calc}/MHz	$\Delta\nu$/kHz
14	7	7	14	6	8	5510.670	5510.666	3.3
12	7	5	12	6	6	5511.477	5511.476	0.9
12	7	6	12	6	7	5511.477	5511.476	0.9
11	7	4	11	6	5	5511.766	5511.760	6.4
11	7	5	11	6	6	5511.766	5511.760	6.4
10	7	3	10	6	4	5511.979	5511.978	0.8
10	7	4	10	6	5	5511.979	5511.978	0.8
9	7	2	9	6	3	5512.150	5512.143	7.2
9	7	3	9	6	4	5512.150	5512.143	7.2
8	7	2	8	6	3	5512.266	5512.263	2.9
8	7	1	8	6	2	5512.266	5512.263	2.9
7	7	0	7	6	1	5512.345	5512.347	-2.9
7	7	1	7	6	2	5512.345	5512.347	-2.9
13	2	12	12	1	11	5569.659	5569.646	12.9
5	5	0	4	4	1	5593.439	5593.438	1.5
5	5	1	4	4	0	5593.439	5593.438	1.5
10	3	8	9	2	7	5646.996	5647.000	-4.1
10	3	7	9	2	8	5689.821	5689.819	1.7
16	1	16	15	0	15	5720.128	5720.129	-1.0
8	4	5	7	3	4	5811.116	5811.128	-12.5
8	4	4	7	3	5	5811.388	5811.380	7.7
14	2	13	13	1	12	5874.033	5874.037	-3.5
12	2	10	11	1	11	5935.519	5935.512	6.2
6	5	1	5	4	2	5948.851	5948.851	-0.5
6	5	2	5	4	1	5948.851	5948.851	-0.4
11	3	9	10	2	8	5988.777	5988.773	3.3

J'	$K_a{}'$	$K_c{}'$	J''	$K_a{}''$	$K_c{}''$	ν_{obs}/MHz	ν_{calc}/MHz	$\Delta\nu$/kHz
17	1	17	16	0	16	6050.128	6050.128	0.6
11	3	8	10	2	9	6052.876	6052.875	1.2
19	1	18	18	2	17	6149.699	6149.698	1.5
9	4	6	8	3	5	6166.029	6166.041	-11.8
9	4	5	8	3	6	6166.604	6166.597	7.5
15	2	14	14	1	13	6175.726	6175.728	-1.6
18	0	18	17	1	17	6228.071	6228.071	0.3
7	5	2	6	4	3	6304.242	6304.246	-3.9
7	5	3	6	4	2	6304.242	6304.245	-3.4
12	3	10	11	2	9	6326.332	6326.331	1.1
22	8	15	22	7	16	6353.777	6353.783	-6.4
22	8	14	22	7	15	6353.777	6353.783	-6.2
20	8	12	20	7	13	6355.880	6355.876	3.4
20	8	13	20	7	14	6355.880	6355.876	3.4
18	8	10	18	7	11	6357.448	6357.438	9.8
18	8	11	18	7	12	6357.448	6357.438	9.8
17	8	10	17	7	11	6358.046	6358.051	-5.3
17	8	9	17	7	10	6358.046	6358.051	-5.3
15	8	8	15	7	9	6358.988	6358.998	-10.9
15	8	7	15	7	8	6358.988	6358.998	-10.9
14	8	6	14	7	7	6359.351	6359.354	-2.6
14	8	7	14	7	8	6359.351	6359.354	-2.6
13	8	6	13	7	7	6359.650	6359.643	7.2
13	8	5	13	7	6	6359.650	6359.643	7.2
12	8	4	12	7	5	6359.877	6359.875	2.7
12	8	5	12	7	6	6359.877	6359.875	2.7

J'	$K_a{}'$	$K_c{}'$	J''	$K_a{}''$	$K_c{}''$	ν_{obs}/MHz	ν_{calc}/MHz	$\Delta\nu$/kHz
10	8	3	10	7	4	6360.200	6360.198	2.0
10	8	2	10	7	3	6360.200	6360.198	2.0
9	8	1	9	7	2	6360.305	6360.304	0.7
9	8	2	9	7	3	6360.305	6360.304	0.7
8	8	1	8	7	2	6360.381	6360.382	-0.7
8	8	0	8	7	1	6360.381	6360.382	-0.7
18	1	18	17	0	17	6382.124	6382.118	5.7
12	3	9	11	2	10	6418.546	6418.545	0.9
16	2	15	15	1	14	6475.122	6475.122	0.1
10	4	7	9	3	6	6520.626	6520.629	-3.3
10	4	6	9	3	7	6521.740	6521.743	-3.1
19	0	19	18	1	18	6588.100	6588.101	-0.5
8	5	3	7	4	4	6659.605	6659.607	-2.3
8	5	4	7	4	3	6659.605	6659.606	-0.8
19	1	19	18	0	18	6716.043	6716.045	-1.6
17	2	16	16	1	15	6772.682	6772.683	-1.6
13	3	10	12	2	11	6787.413	6787.412	1.3
6	6	0	5	5	1	6796.914	6796.911	2.8
6	6	1	5	5	0	6796.914	6796.911	2.8
11	4	8	10	3	7	6874.746	6874.759	-12.6
11	4	7	10	3	8	6876.851	6876.832	19.4
20	0	20	19	1	19	6946.153	6946.159	-6.3
21	1	20	20	2	19	6961.572	6961.583	-11.7
14	3	12	13	2	11	6986.766	6986.761	4.6
9	5	4	8	4	5	7014.914	7014.920	-5.7
9	5	5	8	4	4	7014.914	7014.915	-1.1

J'	$K_a{}'$	$K_c{}'$	J''	$K_a{}''$	$K_c{}''$	ν_{obs}/MHz	ν_{calc}/MHz	$\Delta\nu$/kHz
20	1	20	19	0	19	7051.781	7051.790	-8.3
18	2	17	17	1	16	7068.935	7068.940	-4.3
7	6	1	6	5	2	7152.330	7152.325	5.3
7	6	2	6	5	1	7152.330	7152.325	5.3
14	3	11	13	2	12	7160.126	7160.135	-8.3
21	9	13	21	8	14	7204.711	7204.718	-6.8
21	9	12	21	8	13	7204.711	7204.718	-6.8
14	9	6	14	8	7	7207.751	7207.751	-0.7
14	9	5	14	8	6	7207.751	7207.751	-0.7
12	9	4	12	8	5	7208.114	7208.108	6.8
12	9	3	12	8	4	7208.114	7208.108	6.8
12	4	9	11	3	8	7228.246	7228.261	-15.0
12	4	8	11	3	9	7231.892	7231.893	-1.1
21	0	21	20	1	20	7302.460	7302.454	6.7
15	3	13	14	2	12	7308.892	7308.887	5.2
15	2	13	14	1	14	7312.868	7312.859	8.5
22	1	21	21	2	20	7364.109	7364.100	8.5
19	2	18	18	1	17	7364.468	7364.471	-3.3
10	5	5	9	4	6	7370.162	7370.163	-1.6
21	1	21	20	0	20	7389.185	7389.196	-11.2
8	6	2	7	5	3	7507.725	7507.725	-0.1
8	6	3	7	5	2	7507.725	7507.725	-0.1
15	3	12	14	2	13	7537.442	7537.450	-7.8
13	4	10	12	3	9	7580.925	7580.924	1.0
13	4	9	12	3	10	7586.979	7586.983	-3.8
16	3	14	15	2	13	7625.342	7625.341	0.3

J'	$K_a{}'$	$K_c{}'$	J''	$K_a{}''$	$K_c{}''$	ν_{obs}/MHz	ν_{calc}/MHz	$\Delta\nu$/kHz
22	0	22	21	1	21	7657.189	7657.196	-7.5
20	2	19	19	1	18	7659.902	7659.901	0.8
11	5	7	10	4	6	7725.290	7725.289	1.5
22	1	22	21	0	21	7728.094	7728.088	6.4
9	6	3	8	5	4	7863.100	7863.104	-3.6
9	6	4	8	5	3	7863.100	7863.104	-3.6
16	3	13	15	2	14	7920.174	7920.167	6.5
14	4	11	13	3	10	7932.491	7932.487	3.5
17	3	15	16	2	14	7936.092	7936.083	8.3
14	4	10	13	3	11	7942.177	7942.184	-6.3
21	2	20	20	1	19	7955.871	7955.875	-4.0
7	7	0	6	6	1	8000.367	8000.372	-5.2
7	7	1	6	6	0	8000.367	8000.372	-5.2
23	0	23	22	1	22	8010.604	8010.593	10.8
10	6	4	9	5	5	8218.451	8218.452	-1.3
10	6	5	9	5	4	8218.451	8218.452	-1.2
15	4	12	14	3	11	8282.638	8282.637	0.1
8	7	1	7	6	2	8355.790	8355.783	6.3
8	7	2	7	6	1	8355.790	8355.783	6.3
9	7	2	8	6	3	8711.183	8711.184	-2.0
9	7	3	8	6	2	8711.183	8711.184	-2.0
8	8	0	7	7	1	9203.808	9203.818	-9.5
8	8	1	7	7	0	9203.808	9203.818	-9.5
13	6	7	12	5	8	9284.178	9284.183	-5.0
13	6	8	12	5	7	9284.178	9284.181	-3.8
11	7	4	10	6	5	9421.936	9421.934	2.7

J'	K_a'	K_c'	J''	K_a''	K_c''	ν_{obs}/MHz	ν_{calc}/MHz	$\Delta\nu$/kHz
11	7	5	10	6	4	9421.936	9421.934	2.7
9	8	1	8	7	2	9559.220	9559.226	-5.7
9	8	2	8	7	1	9559.220	9559.226	-5.7

Table S9: Assigned rotational transitions for the ^{208}Pb isotopologue of isomer TEL-TFO-6.

J'	K_a'	K_c'	J''	K_a''	K_c''	ν_{obs}/MHz	ν_{calc}/MHz	$\Delta\nu$/kHz
14	3	12	13	4	9	2014.217	2014.217	0.4
13	3	10	13	2	11	2016.367	2016.363	4.3
12	3	9	12	2	10	2041.304	2041.306	-2.4
11	3	8	11	2	9	2062.195	2062.191	3.5
10	3	7	10	2	8	2079.118	2079.119	-0.8
9	3	6	9	2	7	2092.362	2092.361	0.5
8	3	5	8	2	6	2102.316	2102.317	-0.5
9	1	8	8	2	7	2107.323	2107.321	2.2
12	2	11	11	3	8	2108.101	2108.100	1.1
7	3	4	7	2	5	2109.469	2109.468	1.2
6	3	3	6	2	4	2114.331	2114.329	2.1
5	3	2	5	2	3	2117.415	2117.415	0.4
5	3	3	5	2	4	2121.778	2121.781	-2.7
6	3	4	6	2	5	2123.005	2123.007	-2.1
7	3	5	7	2	6	2124.963	2124.961	2.4
8	3	6	8	2	7	2127.867	2127.865	1.9
9	3	7	9	2	8	2131.964	2131.967	-2.6
7	0	7	6	1	6	2136.854	2136.848	5.5

J'	$K_a{}'$	$K_c{}'$	J''	$K_a{}''$	$K_c{}''$	$\nu_{\rm obs}$/MHz	$\nu_{\rm calc}$/MHz	$\Delta\nu$/kHz
10	3	8	10	2	9	2137.528	2137.528	-0.4
5	1	5	4	0	4	2137.754	2137.752	2.4
11	3	9	11	2	10	2144.825	2144.825	0.9
12	3	10	12	2	11	2154.140	2154.138	1.5
13	3	11	13	2	12	2165.757	2165.755	2.2
14	3	12	14	2	13	2179.959	2179.958	1.3
15	3	13	15	2	14	2197.026	2197.027	-0.2
16	3	14	16	2	15	2217.227	2217.227	-0.4
12	2	10	11	3	9	2230.243	2230.242	1.2
21	2	20	21	1	21	2279.218	2279.217	1.4
19	3	17	19	2	18	2299.047	2299.051	-4.3
22	6	16	21	7	15	2315.411	2315.413	-1.8
22	6	17	21	7	14	2315.411	2315.401	10.8
8	7	1	9	6	4	2316.011	2316.009	2.6
8	7	2	9	6	3	2316.011	2316.009	2.6
3	2	2	2	1	1	2325.279	2325.279	-0.4
20	3	18	20	2	19	2334.108	2334.105	2.4
3	2	1	2	1	2	2351.229	2351.231	-2.4
15	3	13	14	4	10	2370.968	2370.973	-5.6
22	2	21	22	1	22	2372.840	2372.839	0.9
21	3	19	21	2	20	2373.338	2373.340	-2.2
15	3	12	14	4	11	2392.558	2392.561	-2.7
13	2	12	12	3	9	2450.033	2450.032	0.6
23	3	21	23	2	22	2464.844	2464.845	-0.9
6	1	6	5	0	5	2469.150	2469.142	8.4
10	1	9	9	2	8	2501.437	2501.437	-0.5

J'	$K_a{}'$	$K_c{}'$	J''	$K_a{}''$	$K_c{}''$	ν_{obs}/MHz	ν_{calc}/MHz	$\Delta\nu$/kHz
8	0	8	7	1	7	2512.789	2512.788	1.1
12	1	12	11	2	9	2560.082	2560.080	1.5
24	2	23	24	1	24	2569.339	2569.340	-1.2
12	9	4	13	8	5	2591.150	2591.154	-3.2
12	9	3	13	8	6	2591.150	2591.154	-3.2
13	2	11	12	3	10	2614.492	2614.493	-0.9
4	2	3	3	1	2	2667.779	2667.779	0.5
4	2	2	3	1	3	2720.312	2720.310	1.4
16	3	14	15	4	11	2727.469	2727.465	3.9
14	2	13	13	3	10	2788.263	2788.262	1.2
7	1	7	6	0	6	2797.571	2797.571	-0.3
22	4	18	22	3	19	2804.238	2804.242	-3.0
13	1	13	12	2	10	2823.907	2823.901	6.5
20	4	16	20	3	17	2862.035	2862.034	1.0
19	4	15	19	3	16	2884.533	2884.533	-0.1
9	0	9	8	1	8	2889.484	2889.486	-2.2
11	1	10	10	2	9	2898.670	2898.670	0.5
18	4	14	18	3	15	2903.213	2903.213	0.7
17	4	13	17	3	14	2918.494	2918.484	10.2
16	4	12	16	3	13	2930.769	2930.781	-12.3
15	4	11	15	3	12	2940.535	2940.534	1.2
14	4	10	14	3	11	2948.151	2948.150	0.9
13	4	9	13	3	10	2954.006	2954.003	3.0
12	4	8	12	3	9	2958.426	2958.425	1.1
19	4	15	18	5	14	2958.836	2958.828	7.7
17	4	14	17	3	15	2960.075	2960.080	-5.3

J'	$K_a{}'$	$K_c{}'$	J''	$K_a{}''$	$K_c{}''$	ν_{obs}/MHz	ν_{calc}/MHz	$\Delta\nu$/kHz
16	4	13	16	3	14	2960.367	2960.360	6.9
18	4	15	18	3	16	2960.367	2960.390	-22.9
15	4	12	15	3	13	2961.044	2961.046	-1.9
19	4	16	19	3	17	2961.481	2961.497	-15.4
11	4	7	11	3	8	2961.708	2961.705	3.2
14	4	11	14	3	12	2961.984	2961.982	1.7
13	4	10	13	3	11	2963.043	2963.041	1.9
12	4	9	12	3	10	2964.091	2964.120	-29.2
10	4	6	10	3	7	2964.091	2964.087	4.3
11	4	8	11	3	9	2965.144	2965.145	-1.9
9	4	5	9	3	6	2965.783	2965.776	6.6
10	4	7	10	3	8	2966.064	2966.064	-0.1
9	4	6	9	3	7	2966.841	2966.846	-5.5
8	4	4	8	3	5	2966.950	2966.941	9.5
21	4	18	21	3	19	2967.052	2967.052	0.3
7	4	4	7	3	5	2967.959	2967.962	-2.9
6	4	2	6	3	3	2968.218	2968.211	7.1
6	4	3	6	3	4	2968.295	2968.310	-14.5
5	4	2	5	3	3	2968.524	2968.542	-17.6
5	4	1	5	3	2	2968.524	2968.509	15.2
4	4	0	4	3	1	2968.678	2968.675	3.4
4	4	1	4	3	2	2968.678	2968.683	-4.8
22	4	19	22	3	20	2972.024	2972.023	0.7
23	4	20	23	3	21	2978.834	2978.829	4.9
24	4	21	24	3	22	2987.762	2987.761	1.4
14	2	12	13	3	11	3004.240	3004.242	-2.1

J'	K_a'	K_c'	J''	K_a''	K_c''	ν_{obs}/MHz	ν_{calc}/MHz	$\Delta\nu$/kHz
5	2	4	4	1	3	3006.050	3006.051	-0.9
17	3	15	16	4	12	3083.485	3083.483	1.8
5	2	3	4	1	4	3094.852	3094.849	2.5
15	2	14	14	3	11	3122.097	3122.097	-0.1
8	1	8	7	0	7	3123.565	3123.569	-4.6
17	3	14	16	4	13	3128.032	3128.032	0.3
3	3	1	2	2	0	3186.154	3186.153	0.9
3	3	0	2	2	1	3186.279	3186.280	-1.1
10	0	10	9	1	9	3266.262	3266.261	0.1
12	1	11	11	2	10	3298.748	3298.747	1.3
6	2	5	5	1	4	3340.123	3340.122	0.9
15	2	13	14	3	12	3399.616	3399.615	0.2
18	3	16	17	4	13	3438.769	3438.766	2.7
9	1	9	8	0	8	3447.739	3447.735	4.0
6	2	4	5	1	5	3475.477	3475.477	0.4
18	3	15	17	4	14	3500.613	3500.615	-2.0
4	3	2	3	2	1	3541.069	3541.077	-8.4
4	3	1	3	2	2	3541.724	3541.715	9.1
11	0	11	10	1	10	3642.461	3642.461	-0.1
7	2	6	6	1	5	3670.035	3670.035	0.4
13	1	12	12	2	11	3701.347	3701.348	-0.2
25	5	20	25	4	21	3741.903	3741.901	1.1
30	5	26	30	4	27	3758.877	3758.876	1.3
29	5	25	29	4	26	3761.182	3761.181	0.5
23	5	18	23	4	19	3766.570	3766.576	-6.0
10	1	10	9	0	9	3770.713	3770.713	0.1

J'	K_a'	K_c'	J''	K_a''	K_c''	ν_{obs}/MHz	ν_{calc}/MHz	$\Delta\nu$/kHz
26	5	22	26	4	23	3772.019	3772.014	4.9
17	2	16	16	3	13	3773.450	3773.452	-1.7
22	5	17	22	4	18	3775.980	3775.978	2.1
24	5	20	24	4	21	3780.623	3780.623	-0.3
21	5	16	21	4	17	3783.804	3783.805	-1.7
23	5	19	23	4	20	3784.913	3784.909	3.9
22	5	18	22	4	19	3789.043	3789.045	-1.8
19	5	14	19	4	15	3795.673	3795.670	3.1
20	5	16	20	4	17	3796.585	3796.584	0.7
19	5	15	19	4	16	3799.895	3799.897	-1.8
18	5	13	18	4	14	3800.095	3800.093	2.6
16	2	14	15	3	13	3800.618	3800.603	14.6
18	5	14	18	4	15	3802.875	3802.871	3.8
17	5	12	17	4	13	3803.721	3803.720	1.4
17	5	13	17	4	14	3805.498	3805.500	-2.4
16	5	11	16	4	12	3806.679	3806.679	-0.3
16	5	12	16	4	13	3807.791	3807.788	3.2
15	5	10	15	4	11	3809.067	3809.078	-11.3
15	5	11	15	4	12	3809.728	3809.747	-19.3
14	5	9	14	4	10	3811.017	3811.008	8.6
14	5	10	14	4	11	3811.392	3811.397	-5.3
10	5	6	10	4	7	3815.427	3815.442	-14.4
10	5	5	10	4	6	3815.427	3815.414	12.7
9	5	5	9	4	6	3815.955	3815.960	-5.8
9	5	4	9	4	5	3815.955	3815.949	5.8
8	5	3	8	4	4	3816.340	3816.336	4.2

J'	$K_a{'}$	$K_c{'}$	J''	$K_a{''}$	$K_c{''}$	$\nu_{\rm obs}$/MHz	$\nu_{\rm calc}$/MHz	$\Delta\nu$/kHz
8	5	4	8	4	5	3816.340	3816.341	-0.2
7	5	2	7	4	3	3816.610	3816.607	2.5
7	5	3	7	4	4	3816.610	3816.609	1.0
6	5	1	6	4	2	3816.790	3816.788	2.0
6	5	2	6	4	3	3816.790	3816.789	1.6
5	5	0	5	4	1	3816.906	3816.902	3.6
5	5	1	5	4	2	3816.906	3816.902	3.5
7	2	5	6	1	6	3862.930	3862.931	-1.1
19	3	16	18	4	15	3877.171	3877.167	4.8
5	3	3	4	2	2	3895.512	3895.516	-4.4
5	3	2	4	2	3	3897.438	3897.437	0.9
8	2	7	7	1	6	3995.852	3995.853	-1.1
12	0	12	11	1	11	4017.495	4017.496	-1.4
18	2	17	17	3	14	4089.236	4089.235	1.7
11	1	11	10	0	10	4093.163	4093.164	-1.3
14	1	13	13	2	12	4106.101	4106.098	2.5
20	3	18	19	4	15	4145.819	4145.820	-0.4
17	2	15	16	3	14	4207.069	4207.066	3.1
6	3	4	5	2	3	4249.117	4249.121	-4.4
6	3	3	5	2	4	4253.618	4253.618	0.0
8	2	6	7	1	7	4258.037	4258.036	1.0
20	3	17	19	4	16	4258.263	4258.259	4.0
9	2	8	8	1	7	4317.663	4317.664	-0.7
4	4	0	3	3	1	4389.658	4389.657	1.9
4	4	1	3	3	0	4389.658	4389.655	3.0
13	0	13	12	1	12	4390.883	4390.877	6.0

J'	K_a'	K_c'	J''	K_a''	K_c''	ν_{obs}/MHz	ν_{calc}/MHz	$\Delta\nu$/kHz
12	1	12	11	0	11	4415.721	4415.727	-6.1
15	1	14	14	2	13	4512.561	4512.565	-3.9
18	6	13	19	3	16	4582.904	4582.888	16.5
7	3	5	6	2	4	4601.455	4601.457	-2.5
7	3	4	6	2	5	4610.486	4610.480	5.6
18	2	16	17	3	15	4618.757	4618.755	2.4
10	2	9	9	1	8	4635.577	4635.581	-3.9
16	6	10	16	5	11	4660.129	4660.113	16.0
16	6	11	16	5	12	4660.129	4660.135	-6.0
9	2	7	8	1	8	4661.673	4661.672	1.0
14	6	8	14	5	9	4662.188	4662.186	2.5
14	6	9	14	5	10	4662.188	4662.192	-3.3
13	6	8	13	5	9	4662.951	4662.948	3.1
13	6	7	13	5	8	4662.951	4662.945	5.8
12	6	7	12	5	8	4663.552	4663.553	-0.9
12	6	6	12	5	7	4663.552	4663.552	0.3
11	6	5	11	5	6	4664.031	4664.029	1.7
11	6	6	11	5	7	4664.031	4664.030	1.2
10	6	4	10	5	5	4664.395	4664.397	-1.7
10	6	5	10	5	6	4664.395	4664.397	-1.9
9	6	3	9	5	4	4664.672	4664.673	-0.9
9	6	4	9	5	5	4664.672	4664.673	-0.9
8	6	2	8	5	3	4664.875	4664.874	0.7
8	6	3	8	5	4	4664.875	4664.874	0.6
7	6	2	7	5	3	4665.012	4665.016	-3.2
7	6	1	7	5	2	4665.012	4665.016	-3.2

J'	K_a'	K_c'	J''	K_a''	K_c''	ν_{obs}/MHz	ν_{calc}/MHz	$\Delta\nu$/kHz
6	6	0	6	5	1	4665.112	4665.111	0.9
6	6	1	6	5	2	4665.112	4665.111	0.9
13	1	13	12	0	12	4738.985	4738.985	0.2
5	4	2	4	3	1	4744.864	4744.858	6.5
5	4	1	4	3	2	4744.864	4744.866	-1.9
14	0	14	13	1	13	4762.230	4762.234	-3.2
24	4	20	23	5	19	4781.827	4781.827	-0.4
22	3	20	21	4	17	4845.416	4845.410	5.8
16	1	15	15	2	14	4920.256	4920.257	-1.5
8	3	6	7	2	5	4952.014	4952.010	3.3
8	3	5	7	2	6	4968.291	4968.291	-0.6
21	2	20	20	3	17	4985.474	4985.482	-7.9
19	2	17	18	3	16	5035.335	5035.336	-1.0
22	3	19	21	4	18	5036.261	5036.266	-4.9
14	1	14	13	0	13	5063.425	5063.429	-3.8
10	2	8	9	1	9	5074.734	5074.738	-4.6
6	4	3	5	3	2	5100.032	5100.016	16.4
6	4	2	5	3	3	5100.032	5100.049	-16.9
23	3	21	22	4	18	5191.119	5191.121	-1.4
12	2	11	11	1	10	5260.368	5260.368	-0.1
9	3	7	8	2	6	5300.206	5300.203	3.3
9	3	6	8	2	7	5327.373	5327.374	-1.4
17	1	16	16	2	15	5328.623	5328.625	-2.3
15	1	15	14	0	14	5389.436	5389.439	-3.6
7	4	4	6	3	3	5455.097	5455.089	7.4
7	4	3	6	3	4	5455.189	5455.190	-0.7

J'	$K_a{}'$	$K_c{}'$	J''	$K_a{}''$	$K_c{}''$	$\nu_{\rm obs}$/MHz	$\nu_{\rm calc}$/MHz	$\Delta\nu$/kHz
16	0	16	15	1	15	5498.032	5498.035	-3.2
11	2	9	10	1	10	5498.112	5498.108	4.0
23	7	17	23	6	18	5500.977	5500.986	-9.3
23	7	16	23	6	17	5500.977	5500.965	11.7
22	7	16	22	6	17	5502.971	5502.978	-6.8
22	7	15	22	6	16	5502.971	5502.966	5.5
20	7	14	20	6	15	5506.229	5506.234	-5.3
20	7	13	20	6	14	5506.229	5506.230	-1.3
19	7	13	19	6	14	5507.539	5507.541	-1.5
19	7	12	19	6	13	5507.539	5507.539	0.7
18	7	11	18	6	12	5508.664	5508.660	4.7
18	7	12	18	6	13	5508.664	5508.661	3.6
17	7	11	17	6	12	5509.612	5509.613	-0.4
17	7	10	17	6	11	5509.612	5509.612	0.2
16	7	9	16	6	10	5510.416	5510.414	1.9
16	7	10	16	6	11	5510.416	5510.415	1.7
15	7	8	15	6	9	5511.089	5511.083	6.5
15	7	9	15	6	10	5511.089	5511.083	6.4
14	7	8	14	6	9	5511.636	5511.633	2.4
14	7	7	14	6	8	5511.636	5511.633	2.5
13	7	6	13	6	7	5512.081	5512.081	0.1
13	7	7	13	6	8	5512.081	5512.081	0.1
12	7	6	12	6	7	5512.441	5512.440	1.5
12	7	5	12	6	6	5512.441	5512.440	1.5
11	7	4	11	6	5	5512.723	5512.722	1.3
11	7	5	11	6	6	5512.723	5512.722	1.3

J'	K_a'	K_c'	J''	K_a''	K_c''	ν_{obs}/MHz	ν_{calc}/MHz	$\Delta\nu$/kHz
10	7	3	10	6	4	5512.942	5512.940	2.1
10	7	4	10	6	5	5512.942	5512.940	2.1
9	7	2	9	6	3	5513.105	5513.103	1.5
9	7	3	9	6	4	5513.105	5513.103	1.5
8	7	1	8	6	2	5513.224	5513.223	1.5
8	7	2	8	6	3	5513.224	5513.223	1.5
7	7	1	7	6	2	5513.309	5513.307	2.0
7	7	0	7	6	1	5513.309	5513.307	2.0
13	2	12	12	1	11	5567.658	5567.659	-0.5
5	5	1	4	4	0	5593.086	5593.085	0.9
5	5	0	4	4	1	5593.086	5593.085	0.9
10	3	8	9	2	7	5645.406	5645.417	-10.4
10	3	7	9	2	8	5688.112	5688.109	3.1
16	1	16	15	0	15	5717.280	5717.272	7.8
18	1	17	17	2	16	5737.069	5737.071	-2.0
8	4	5	7	3	4	5810.010	5810.021	-11.2
8	4	4	7	3	5	5810.284	5810.272	12.1
17	0	17	16	1	16	5862.364	5862.358	6.5
14	2	13	13	1	12	5871.914	5871.908	5.4
6	5	1	5	4	2	5948.295	5948.295	-0.2
6	5	2	5	4	1	5948.295	5948.295	-0.2
11	3	9	10	2	8	5987.026	5987.025	0.7
17	1	17	16	0	16	6047.061	6047.064	-2.7
11	3	8	10	2	9	6050.942	6050.938	3.6
19	1	18	18	2	17	6144.961	6144.957	3.9
9	4	6	8	3	5	6164.722	6164.732	-9.4

J'	$K_a{}'$	$K_c{}'$	J''	$K_a{}''$	$K_c{}''$	ν_{obs}/MHz	ν_{calc}/MHz	$\Delta\nu$/kHz
9	4	5	8	3	6	6165.293	6165.285	7.7
15	2	14	14	1	13	6173.458	6173.458	0.5
18	0	18	17	1	17	6224.373	6224.376	-3.2
7	5	3	6	4	2	6303.488	6303.487	0.8
7	5	2	6	4	3	6303.488	6303.487	0.4
12	3	10	11	2	9	6324.426	6324.429	-3.1
25	8	18	25	7	19	6350.559	6350.556	3.0
25	8	17	25	7	18	6350.559	6350.555	4.2
24	8	16	24	7	17	6352.193	6352.195	-2.2
24	8	17	24	7	18	6352.193	6352.196	-2.9
23	8	15	23	7	16	6353.642	6353.645	-2.3
23	8	16	23	7	17	6353.642	6353.645	-2.7
21	8	13	21	7	14	6356.036	6356.035	0.1
21	8	14	21	7	15	6356.036	6356.036	0.0
20	8	13	20	7	14	6357.006	6357.005	0.6
20	8	12	20	7	13	6357.006	6357.005	0.6
16	8	8	16	7	9	6359.676	6359.686	-9.7
16	8	9	16	7	10	6359.676	6359.686	-9.7
15	8	7	15	7	8	6360.097	6360.114	-17.2
15	8	8	15	7	9	6360.097	6360.114	-17.2
14	8	7	14	7	8	6360.469	6360.468	1.0
14	8	6	14	7	7	6360.469	6360.468	1.0
13	8	5	13	7	6	6360.757	6360.756	0.8
13	8	6	13	7	7	6360.757	6360.756	0.8
12	8	5	12	7	6	6360.989	6360.986	2.4
12	8	4	12	7	5	6360.989	6360.986	2.4

J'	$K_a{}'$	$K_c{}'$	J''	$K_a{}''$	$K_c{}''$	ν_{obs}/MHz	ν_{calc}/MHz	$\Delta\nu$/kHz
11	8	3	11	7	4	6361.171	6361.168	3.2
11	8	4	11	7	5	6361.171	6361.168	3.2
10	8	2	10	7	3	6361.313	6361.308	5.0
10	8	3	10	7	4	6361.313	6361.308	5.0
9	8	2	9	7	3	6361.415	6361.413	1.8
9	8	1	9	7	2	6361.415	6361.413	1.8
8	8	0	8	7	1	6361.497	6361.490	7.0
8	8	1	8	7	2	6361.497	6361.490	7.0
18	1	18	17	0	17	6378.856	6378.844	11.5
13	2	11	12	1	12	6378.856	6378.842	13.7
12	3	9	11	2	10	6416.372	6416.373	-0.6
16	2	15	15	1	14	6472.711	6472.708	2.7
10	4	7	9	3	6	6519.119	6519.120	-0.5
10	4	6	9	3	7	6520.229	6520.229	-0.0
20	1	19	19	2	18	6551.631	6551.629	1.3
13	3	11	12	2	10	6657.094	6657.093	0.6
8	5	4	7	4	3	6658.647	6658.645	2.6
8	5	3	7	4	4	6658.647	6658.646	1.1
19	1	19	18	0	18	6712.558	6712.559	-1.3
13	3	10	12	2	11	6784.988	6784.994	-5.6
6	6	0	5	5	1	6796.506	6796.504	2.0
6	6	1	5	5	0	6796.506	6796.504	2.0
11	4	7	10	3	8	6875.113	6875.115	-1.9
20	0	20	19	1	19	6942.123	6942.124	-1.0
21	1	20	20	2	19	6956.436	6956.440	-4.1
14	3	12	13	2	11	6984.575	6984.583	-7.6

J'	$K_a{}'$	$K_c{}'$	J''	$K_a{}''$	$K_c{}''$	ν_{obs}/MHz	ν_{calc}/MHz	$\Delta\nu$/kHz
9	5	5	8	4	4	7013.750	7013.751	-1.3
9	5	4	8	4	5	7013.750	7013.756	-5.9
20	1	20	19	0	19	7048.097	7048.092	5.0
18	2	17	17	1	16	7066.210	7066.228	-18.4
7	6	1	6	5	2	7151.722	7151.714	8.1
7	6	2	6	5	1	7151.722	7151.714	8.1
14	3	11	13	2	12	7157.456	7157.459	-3.2
12	4	9	11	3	8	7226.359	7226.358	1.0
12	4	8	11	3	9	7229.974	7229.974	0.6
21	0	21	20	1	20	7298.248	7298.247	1.3
15	3	13	14	2	12	7306.590	7306.586	4.2
15	2	13	14	1	14	7308.730	7308.734	-3.6
22	1	21	21	2	20	7358.776	7358.776	0.8
19	2	18	18	1	17	7361.605	7361.601	4.4
10	5	5	9	4	6	7368.800	7368.797	3.0
10	5	6	9	4	5	7368.800	7368.785	14.9
21	1	21	20	0	20	7385.283	7385.288	-4.1
8	6	3	7	5	2	7506.911	7506.912	-0.5
8	6	2	7	5	3	7506.911	7506.912	-0.5
15	3	12	14	2	13	7534.496	7534.501	-5.0
13	4	10	12	3	9	7578.827	7578.828	-0.3
13	4	9	12	3	10	7584.844	7584.859	-15.2
16	3	14	15	2	13	7622.940	7622.927	13.0
22	0	22	21	1	21	7652.821	7652.817	4.1
20	2	19	19	1	18	7656.863	7656.863	-0.2
11	5	7	10	4	6	7723.717	7723.720	-3.4

J'	$K_a{}'$	$K_c{}'$	J''	$K_a{}''$	$K_c{}''$	ν_{obs}/MHz	ν_{calc}/MHz	$\Delta\nu$/kHz
23	1	22	22	2	21	7758.088	7758.089	-0.4
16	2	14	15	1	15	7792.951	7792.950	0.7
9	6	3	8	5	4	7862.084	7862.088	-3.6
9	6	4	8	5	3	7862.084	7862.088	-3.6
16	3	13	15	2	14	7916.915	7916.929	-13.8
14	4	11	13	3	10	7930.203	7930.202	0.3
17	3	15	16	2	14	7933.562	7933.563	-1.2
14	4	10	13	3	11	7939.862	7939.855	7.3
7	7	1	6	6	0	7999.907	7999.910	-2.8
7	7	0	6	6	1	7999.907	7999.910	-2.8
10	6	5	9	5	4	8217.234	8217.233	0.4
10	6	4	9	5	5	8217.234	8217.233	0.4
8	7	2	7	6	1	8355.109	8355.119	-9.5
8	7	1	7	6	2	8355.109	8355.119	-9.5
12	6	6	11	5	7	8927.374	8927.382	-8.1
12	6	7	11	5	6	8927.374	8927.382	-7.6
10	7	3	9	6	4	9065.505	9065.500	4.7
10	7	4	9	6	3	9065.505	9065.500	4.7
8	8	1	7	7	0	9203.294	9203.302	-8.7
8	8	0	7	7	1	9203.294	9203.302	-8.7
13	6	7	12	5	8	9282.368	9282.357	10.9
13	6	8	12	5	7	9282.368	9282.356	12.1

3.2 Frequency lists of TEL-TFO-Ne complexes.

Table S10: Rotational transitions for the ^{206}Pb^{20}Ne isotopologue of the assigned TEL-TFO-Ne trimer.

J'	$K_a{}'$	$K_c{}'$	J''	$K_a{}''$	$K_c{}''$	ν_{obs}/MHz	ν_{calc}/MHz	$\Delta\nu$/kHz
5	2	4	4	1	4	2573.278	2573.270	7.4
6	2	5	5	1	5	2911.704	2911.703	1.8
7	2	5	6	1	5	3131.800	3131.802	-1.8
5	3	2	4	2	2	3159.858	3159.861	-3.2
5	3	3	4	2	3	3161.304	3161.310	-5.9
15	6	9	15	5	11	3402.373	3402.377	-4.5
15	6	10	15	5	10	3402.373	3402.367	5.8
14	6	9	14	5	9	3403.116	3403.109	6.9
14	6	8	14	5	10	3403.116	3403.114	1.8
13	6	7	13	5	9	3403.718	3403.715	3.1
13	6	8	13	5	8	3403.718	3403.712	5.5
11	6	6	11	5	6	3404.575	3404.576	-1.3
11	6	5	11	5	7	3404.575	3404.577	-1.8
10	6	5	10	5	5	3404.875	3404.871	4.1
10	6	4	10	5	6	3404.875	3404.871	4.0
9	6	3	9	5	5	3405.092	3405.093	-1.0
9	6	4	9	5	4	3405.092	3405.093	-1.0
4	4	1	3	3	1	3457.374	3457.368	5.5
4	4	0	3	3	0	3457.374	3457.367	6.4
6	3	3	5	2	3	3481.193	3481.196	-3.4
6	3	4	5	2	4	3484.546	3484.555	-8.5
9	2	7	8	1	7	3748.925	3748.918	6.6

J'	$K_a{}'$	$K_c{}'$	J''	$K_a{}''$	$K_c{}''$	ν_{obs}/MHz	ν_{calc}/MHz	$\Delta\nu$/kHz
16	2	14	15	3	12	3779.615	3779.616	-1.0
5	4	1	4	3	1	3779.902	3779.898	3.8
5	4	2	4	3	2	3779.902	3779.905	-2.8
7	3	4	6	2	4	3801.597	3801.605	-8.4
7	3	5	6	2	5	3808.264	3808.265	-1.6
14	7	7	14	6	9	4023.232	4023.230	1.9
14	7	8	14	6	8	4023.232	4023.230	2.0
13	7	6	13	6	8	4023.595	4023.591	4.6
13	7	7	13	6	7	4023.595	4023.591	4.6
11	7	4	11	6	6	4024.107	4024.110	-3.2
11	7	5	11	6	5	4024.107	4024.110	-3.2
10	7	4	10	6	4	4024.289	4024.288	0.7
10	7	3	10	6	5	4024.289	4024.288	0.7
9	7	2	9	6	4	4024.425	4024.424	1.5
9	7	3	9	6	3	4024.425	4024.424	1.5
15	0	15	14	1	13	4037.415	4037.419	-4.4
8	3	5	7	2	5	4120.731	4120.739	-7.5
8	3	6	7	2	6	4132.598	4132.600	-2.1
11	2	9	10	1	9	4370.266	4370.258	8.0
5	5	1	4	4	1	4399.086	4399.086	0.3
5	5	0	4	4	0	4399.086	4399.086	0.3
9	3	7	8	2	7	4457.732	4457.734	-2.3
21	8	13	21	7	15	4639.280	4639.281	-0.3
21	8	14	21	7	14	4639.280	4639.280	-0.2
19	8	11	19	7	13	4640.731	4640.733	-2.6
19	8	12	19	7	12	4640.731	4640.733	-2.5

J'	$K_a{}'$	$K_c{}'$	J''	$K_a{}''$	$K_c{}''$	ν_{obs}/MHz	ν_{calc}/MHz	$\Delta\nu$/kHz
17	8	9	17	7	11	4641.800	4641.806	-5.7
17	8	10	17	7	10	4641.800	4641.806	-5.7
16	8	8	16	7	10	4642.220	4642.223	-3.1
16	8	9	16	7	9	4642.220	4642.223	-3.1
14	8	7	14	7	7	4642.865	4642.862	3.5
14	8	6	14	7	8	4642.865	4642.862	3.5
12	8	5	12	7	5	4643.277	4643.290	-12.8
12	8	4	12	7	6	4643.277	4643.290	-12.8
11	8	3	11	7	5	4643.446	4643.442	4.6
11	8	4	11	7	4	4643.446	4643.442	4.6
9	8	2	9	7	2	4643.651	4643.651	0.1
9	8	1	9	7	3	4643.651	4643.651	0.1
12	2	10	11	1	10	4684.437	4684.428	8.9
10	3	7	9	2	7	4753.698	4753.703	-5.7
10	3	8	9	2	8	4783.856	4783.855	0.7
13	2	11	12	1	11	5001.904	5001.891	13.0
7	5	3	6	4	3	5044.137	5044.141	-4.2
7	5	2	6	4	2	5044.137	5044.141	-3.9
11	3	8	10	2	8	5066.835	5066.838	-2.8
9	4	5	8	3	5	5069.296	5069.284	11.4
9	4	6	8	3	6	5069.696	5069.713	-16.4
11	3	9	10	2	9	5111.163	5111.161	2.2
14	2	12	13	1	12	5323.299	5323.292	6.4
6	6	1	5	5	1	5340.784	5340.785	-1.3
6	6	0	5	5	0	5340.784	5340.785	-1.3
8	5	3	7	4	3	5366.629	5366.633	-3.7

J'	$K_a{}'$	$K_c{}'$	J''	$K_a{}''$	$K_c{}''$	ν_{obs}/MHz	ν_{calc}/MHz	$\Delta\nu$/kHz
8	5	4	7	4	4	5366.629	5366.634	-5.0
10	4	6	9	3	6	5391.184	5391.178	5.3
10	4	7	9	3	7	5392.025	5392.031	-5.9
12	3	10	11	2	10	5439.847	5439.854	-7.3
9	5	4	8	4	4	5689.091	5689.084	7.0
9	5	5	8	4	5	5689.091	5689.088	3.3
11	4	8	10	3	8	5714.296	5714.295	1.4
13	3	11	12	2	11	5770.149	5770.141	7.9
16	2	14	15	1	14	5980.135	5980.148	-12.8
8	6	3	7	5	3	5985.833	5985.841	-7.2
8	6	2	7	5	2	5985.833	5985.841	-7.2
10	5	5	9	4	5	6011.482	6011.477	5.4
10	5	6	9	4	6	6011.482	6011.486	-4.2
12	4	8	11	3	8	6033.791	6033.787	3.6
12	4	9	11	3	9	6036.516	6036.521	-4.8
14	3	12	13	2	12	6102.213	6102.226	-13.5
7	7	0	6	6	0	6282.470	6282.464	6.2
7	7	1	6	6	1	6282.470	6282.464	6.2
9	6	3	8	5	3	6308.346	6308.345	1.5
9	6	4	8	5	4	6308.346	6308.345	1.5
13	4	9	12	3	9	6354.223	6354.222	1.5
15	3	13	14	2	13	6436.306	6436.311	-5.1
8	7	2	7	6	2	6604.989	6604.993	-3.3
8	7	1	7	6	1	6604.989	6604.993	-3.3
10	6	4	9	5	4	6630.824	6630.823	0.8
10	6	5	9	5	5	6630.824	6630.823	0.8

J'	$K_a{}'$	$K_c{}'$	J''	$K_a{}''$	$K_c{}''$	ν_{obs}/MHz	ν_{calc}/MHz	$\Delta\nu$/kHz
14	4	10	13	3	10	6673.827	6673.840	-13.1
14	4	11	13	3	11	6681.012	6681.000	12.2
9	7	2	8	6	2	6927.516	6927.512	3.8
9	7	3	8	6	3	6927.516	6927.512	3.8
11	6	6	10	5	6	6953.274	6953.266	7.8
11	6	5	10	5	5	6953.274	6953.266	8.0
15	4	12	14	3	12	7003.378	7003.363	15.2
8	8	1	7	7	1	7224.121	7224.117	4.1

Table S11: Rotational transitions for the ^{206}Pb^{22}Ne isotopologue of the assigned TEL-TFO-Ne trimer.

J'	$K_a{}'$	$K_c{}'$	J''	$K_a{}''$	$K_c{}''$	ν_{obs}/MHz	ν_{calc}/MHz	$\Delta\nu$/kHz
5	3	2	4	2	2	3110.106	3110.119	-12.5
14	6	8	14	5	10	3324.779	3324.787	-7.5
14	6	9	14	5	9	3324.779	3324.777	2.7
13	6	8	13	5	8	3325.569	3325.561	8.3
13	6	7	13	5	9	3325.569	3325.565	3.5
12	6	7	12	5	7	3326.187	3326.188	-0.8
12	6	6	12	5	8	3326.187	3326.190	-3.0
11	6	5	11	5	7	3326.677	3326.682	-4.5
11	6	6	11	5	6	3326.677	3326.681	-3.7
9	6	4	9	5	4	3327.351	3327.348	3.2
9	6	3	9	5	5	3327.351	3327.348	3.1
8	6	2	8	5	4	3327.553	3327.558	-5.1

J'	$K_a{}'$	$K_c{}'$	J''	$K_a{}''$	$K_c{}''$	ν_{obs}/MHz	ν_{calc}/MHz	$\Delta\nu$/kHz
8	6	3	8	5	3	3327.553	3327.558	-5.1
4	4	1	3	3	1	3396.613	3396.608	4.8
4	4	0	3	3	0	3396.613	3396.607	6.2
5	4	1	4	3	1	3716.299	3716.294	4.9
5	4	2	4	3	2	3716.299	3716.304	-4.9
7	3	4	6	2	4	3745.222	3745.215	7.0
10	7	4	10	6	4	3932.447	3932.448	-1.2
10	7	3	10	6	5	3932.447	3932.448	-1.2
8	3	5	7	2	5	4060.534	4060.545	-11.2
5	5	0	4	4	0	4321.371	4321.372	-1.0
5	5	1	4	4	1	4321.371	4321.372	-1.0
9	3	6	8	2	6	4373.815	4373.804	10.2
6	5	2	5	4	2	4641.068	4641.067	0.9
6	5	1	5	4	1	4641.068	4641.067	1.0
10	3	7	9	2	7	4684.569	4684.572	-2.8
7	5	2	6	4	2	4960.744	4960.741	2.5
7	5	3	6	4	3	4960.744	4960.742	1.9
9	4	5	8	3	5	4994.052	4994.053	-0.8
9	4	6	8	3	6	4994.684	4994.691	-7.1
6	6	0	5	5	0	5246.128	5246.119	9.5
6	6	1	5	5	1	5246.128	5246.119	9.5
8	5	4	7	4	4	5280.383	5280.384	-1.1
8	5	3	7	4	3	5280.383	5280.382	1.0
10	4	6	9	3	6	5312.874	5312.876	-2.3
10	4	7	9	3	7	5314.145	5314.146	-1.4
7	6	2	6	5	2	5565.811	5565.812	-1.1

J'	$K_a{}'$	$K_c{}'$	J''	$K_a{}''$	$K_c{}''$	ν_{obs}/MHz	ν_{calc}/MHz	$\Delta\nu$/kHz
7	6	1	6	5	1	5565.811	5565.812	-1.1
9	5	4	8	4	4	5599.970	5599.969	0.3
9	5	5	8	4	5	5599.970	5599.976	-6.0
8	6	3	7	5	3	5885.501	5885.492	9.1
8	6	2	7	5	2	5885.501	5885.492	9.1
12	4	9	11	3	9	5952.959	5952.958	1.6
7	7	1	6	6	1	6170.846	6170.845	1.8
7	7	0	6	6	0	6170.846	6170.845	1.8
9	6	4	8	5	4	6205.143	6205.149	-5.8
9	6	3	8	5	3	6205.143	6205.149	-5.8
8	7	2	7	6	2	6490.532	6490.534	-1.6
8	7	1	7	6	1	6490.532	6490.534	-1.6
10	6	5	9	5	5	6524.779	6524.775	3.8
10	6	4	9	5	4	6524.779	6524.775	3.9
9	7	2	8	6	2	6810.211	6810.213	-1.9
9	7	3	8	6	3	6810.211	6810.213	-1.9
11	6	6	10	5	6	6844.361	6844.357	4.7
11	6	5	10	5	5	6844.361	6844.356	5.0
8	8	0	7	7	0	7095.541	7095.546	-5.2
8	8	1	7	7	1	7095.541	7095.546	-5.2
12	6	6	11	5	6	7163.880	7163.880	0.1
12	6	7	11	5	7	7163.880	7163.881	-0.8
9	8	2	8	7	2	7415.230	7415.230	0.3
9	8	1	8	7	1	7415.230	7415.230	0.3
11	7	4	10	6	4	7449.511	7449.515	-3.3
11	7	5	10	6	5	7449.511	7449.515	-3.4

J'	K_a'	K_c'	J''	K_a''	K_c''	ν_{obs}/MHz	ν_{calc}/MHz	$\Delta\nu$/kHz
13	6	8	12	5	8	7483.337	7483.332	4.5
13	6	7	12	5	7	7483.337	7483.330	6.5
10	8	2	9	7	2	7734.907	7734.905	2.1
10	8	3	9	7	3	7734.907	7734.905	2.1
12	7	6	11	6	6	7769.126	7769.123	2.1
12	7	5	11	6	5	7769.126	7769.123	2.1
14	6	9	13	5	9	7802.687	7802.695	-7.2
14	6	8	13	5	8	7802.687	7802.690	-2.6

Table S12: Rotational transitions for the ^{207}Pb^{20}Ne isotopologue of the assigned TEL-TFO-Ne trimer.

J'	K_a'	K_c'	J''	K_a''	K_c''	ν_{obs}/MHz	ν_{calc}/MHz	$\Delta\nu$/kHz
12	1	11	11	2	9	3104.324	3104.321	2.9
7	2	5	6	1	5	3130.700	3130.699	1.4
5	3	2	4	2	2	3159.293	3159.294	-1.2
5	3	3	4	2	3	3160.739	3160.740	-1.1
9	1	8	8	0	8	3366.216	3366.208	8.1
4	4	1	3	3	1	3457.173	3457.172	0.4
4	4	0	3	3	0	3457.173	3457.171	1.3
6	3	3	5	2	3	3480.432	3480.432	-0.1
6	3	4	5	2	4	3483.781	3483.784	-3.2
9	2	7	8	1	7	3747.430	3747.426	3.4
5	4	1	4	3	1	3779.508	3779.502	6.2
5	4	2	4	3	2	3779.508	3779.509	-0.4

J'	$K_a{}'$	$K_c{}'$	J''	$K_a{}''$	$K_c{}''$	ν_{obs}/MHz	ν_{calc}/MHz	$\Delta\nu$/kHz
7	3	4	6	2	4	3800.627	3800.645	-18.3
7	3	5	6	2	5	3807.296	3807.294	2.6
16	7	10	16	6	10	4023.374	4023.373	1.1
16	7	9	16	6	11	4023.374	4023.373	0.8
13	7	7	13	6	7	4024.717	4024.712	4.9
13	7	6	13	6	8	4024.717	4024.712	4.9
11	7	5	11	6	5	4025.232	4025.233	-1.2
11	7	4	11	6	6	4025.232	4025.233	-1.2
9	7	3	9	6	3	4025.544	4025.548	-4.3
9	7	2	9	6	4	4025.544	4025.548	-4.3
8	7	1	8	6	3	4025.651	4025.649	2.7
8	7	2	8	6	2	4025.651	4025.649	2.7
7	7	1	7	6	1	4025.727	4025.721	6.2
7	7	0	7	6	2	4025.727	4025.721	6.2
10	2	8	9	1	8	4056.983	4056.981	2.6
8	3	5	7	2	5	4119.589	4119.587	1.4
8	3	6	7	2	6	4131.429	4131.426	2.8
10	2	9	9	1	9	4295.756	4295.763	-7.0
11	2	9	10	1	9	4368.376	4368.359	16.7
5	5	1	4	4	1	4398.861	4398.863	-1.5
5	5	0	4	4	0	4398.861	4398.863	-1.5
9	3	7	8	2	7	4456.359	4456.357	2.0
17	8	9	17	7	11	4643.100	4643.098	2.4
17	8	10	17	7	10	4643.100	4643.098	2.4
12	8	5	12	7	5	4644.585	4644.586	-1.8
12	8	4	12	7	6	4644.585	4644.586	-1.8

J'	$K_a{}'$	$K_c{}'$	J''	$K_a{}''$	$K_c{}''$	ν_{obs}/MHz	ν_{calc}/MHz	$\Delta\nu$/kHz
11	8	3	11	7	5	4644.731	4644.740	-8.4
11	8	4	11	7	4	4644.731	4644.740	-8.4
10	8	2	10	7	4	4644.865	4644.859	6.0
10	8	3	10	7	3	4644.865	4644.859	6.0
9	8	1	9	7	3	4644.955	4644.951	3.9
9	8	2	9	7	2	4644.955	4644.951	3.9
8	8	1	8	7	1	4645.019	4645.020	-1.6
8	8	0	8	7	2	4645.019	4645.020	-1.6
12	2	10	11	1	10	4682.306	4682.314	-7.3
6	5	1	5	4	1	4721.206	4721.199	7.3
6	5	2	5	4	2	4721.206	4721.199	7.3
8	4	4	7	3	4	4746.157	4746.147	10.4
8	4	5	7	3	5	4746.326	4746.342	-16.0
10	3	7	9	2	7	4752.184	4752.181	2.1
13	2	11	12	1	11	4999.553	4999.550	2.8
7	5	3	6	4	3	5043.518	5043.519	-1.3
7	5	2	6	4	2	5043.518	5043.519	-0.9
11	3	8	10	2	8	5065.138	5065.140	-1.8
9	4	6	8	3	6	5068.508	5068.521	-13.1
11	3	9	10	2	9	5109.369	5109.370	-0.7
13	9	5	13	8	5	5263.889	5263.896	-6.8
13	9	4	13	8	6	5263.889	5263.896	-6.8
14	2	12	13	1	12	5320.696	5320.711	-15.7
6	6	0	5	5	0	5340.539	5340.537	2.4
6	6	1	5	5	1	5340.539	5340.537	2.4
8	5	4	7	4	4	5365.803	5365.813	-9.9

J'	$K_a{}'$	$K_c{}'$	J''	$K_a{}''$	$K_c{}''$	ν_{obs}/MHz	ν_{calc}/MHz	$\Delta\nu$/kHz
8	5	3	7	4	3	5365.803	5365.812	-8.7
12	3	9	11	2	9	5375.508	5375.507	0.9
10	4	6	9	3	6	5389.796	5389.792	3.9
10	4	7	9	3	7	5390.637	5390.642	-4.4
7	6	1	6	5	1	5662.874	5662.870	3.6
7	6	2	6	5	2	5662.874	5662.870	3.6
13	3	10	12	2	10	5683.137	5683.139	-1.6
11	4	8	10	3	8	5712.714	5712.709	5.2
16	2	14	15	1	14	5977.047	5977.038	8.6
8	6	2	7	5	2	5985.189	5985.193	-4.2
8	6	3	7	5	3	5985.189	5985.193	-4.2
14	3	11	13	2	11	5988.026	5988.031	-5.1
10	5	5	9	4	5	6010.266	6010.260	6.1
10	5	6	9	4	6	6010.266	6010.269	-3.5
12	4	8	11	3	8	6032.020	6032.014	6.1
12	4	9	11	3	9	6034.732	6034.739	-7.0
7	7	1	6	6	1	6282.194	6282.190	4.4
7	7	0	6	6	0	6282.194	6282.190	4.4
15	3	12	14	2	12	6290.348	6290.343	4.9
9	6	4	8	5	4	6307.501	6307.498	3.1
9	6	3	8	5	3	6307.501	6307.498	3.1
17	2	15	16	1	15	6313.158	6313.161	-3.0
11	5	7	10	4	7	6332.395	6332.399	-4.1
11	5	6	10	4	6	6332.395	6332.376	18.2
8	7	2	7	6	2	6604.514	6604.519	-5.0
8	7	1	7	6	1	6604.514	6604.519	-5.0

J'	$K_a{}'$	$K_c{}'$	J''	$K_a{}''$	$K_c{}''$	ν_{obs}/MHz	ν_{calc}/MHz	$\Delta\nu$/kHz
10	6	4	9	5	4	6629.778	6629.778	-0.0
10	6	5	9	5	5	6629.778	6629.778	-0.1
14	4	10	13	3	10	6671.687	6671.692	-4.4
14	4	11	13	3	11	6678.828	6678.827	1.8
9	7	2	8	6	2	6926.843	6926.840	3.2
9	7	3	8	6	3	6926.843	6926.840	3.2
11	6	5	10	5	5	6952.033	6952.024	8.9
11	6	6	10	5	6	6952.033	6952.024	8.8
8	8	1	7	7	1	7223.818	7223.819	-0.4
8	8	0	7	7	0	7223.818	7223.819	-0.4
10	7	3	9	6	3	7249.145	7249.147	-2.6
10	7	4	9	6	4	7249.145	7249.147	-2.6
12	6	7	11	5	7	7274.222	7274.225	-2.1
12	6	6	11	5	6	7274.222	7274.224	-1.7
9	8	2	8	7	2	7546.144	7546.143	1.2
9	8	1	8	7	1	7546.144	7546.143	1.2
11	7	4	10	6	4	7571.435	7571.436	-1.3
11	7	5	10	6	5	7571.435	7571.436	-1.3

Table S13: Rotational transitions for the ^{207}Pb^{22}Ne isotopologue of the assigned TEL-TFO-Ne trimer.

J'	$K_a{}'$	$K_c{}'$	J''	$K_a{}''$	$K_c{}''$	ν_{obs}/MHz	ν_{calc}/MHz	$\Delta\nu$/kHz
5	3	2	4	2	2	3109.568	3109.562	5.9
15	6	9	15	5	11	3324.779	3324.792	-13.2

J'	K_a'	K_c'	J''	K_a''	K_c''	ν_{obs}/MHz	ν_{calc}/MHz	$\Delta\nu$/kHz
15	6	10	15	5	10	3324.779	3324.772	7.1
11	6	5	11	5	7	3327.641	3327.641	-0.4
11	6	6	11	5	6	3327.641	3327.641	0.5
8	2	6	7	1	6	3389.692	3389.692	0.4
4	4	1	3	3	1	3396.418	3396.425	-7.4
4	4	0	3	3	0	3396.418	3396.424	-6.0
6	3	3	5	2	3	3427.508	3427.508	-0.8
10	2	8	9	1	8	4003.006	4003.007	-0.3
5	5	0	4	4	0	4321.159	4321.163	-4.6
5	5	1	4	4	1	4321.159	4321.163	-4.6
16	1	15	15	2	13	4367.977	4367.977	-0.7
9	3	6	8	2	6	4372.488	4372.486	1.9
6	5	1	5	4	1	4640.654	4640.657	-2.7
6	5	2	5	4	2	4640.654	4640.657	-2.8
10	3	7	9	2	7	4683.077	4683.078	-0.7
7	5	3	6	4	3	4960.130	4960.132	-2.2
7	5	2	6	4	2	4960.130	4960.132	-1.7
9	4	6	8	3	6	4993.492	4993.509	-16.5
6	6	1	5	5	1	5245.893	5245.883	9.7
6	6	0	5	5	0	5245.893	5245.883	9.7
14	2	12	13	1	12	5268.278	5268.279	-1.0
8	5	4	7	4	4	5279.570	5279.574	-4.0
8	5	3	7	4	3	5279.570	5279.572	-1.9
10	4	7	9	3	7	5312.767	5312.765	2.2
7	6	1	6	5	1	5565.381	5565.375	5.8
7	6	2	6	5	2	5565.381	5565.375	5.8

J'	$K_a{}'$	$K_c{}'$	J''	$K_a{}''$	$K_c{}''$	ν_{obs}/MHz	ν_{calc}/MHz	$\Delta\nu$/kHz
9	5	4	8	4	4	5598.971	5598.959	11.2
9	5	5	8	4	5	5598.971	5598.966	4.8
11	4	7	10	3	7	5629.653	5629.644	8.7
11	4	8	10	3	8	5631.971	5631.977	-5.8
12	4	8	11	3	8	5947.127	5947.132	-5.0
12	4	9	11	3	9	5951.184	5951.179	4.9
7	7	0	6	6	0	6170.586	6170.581	5.0
7	7	1	6	6	1	6170.586	6170.581	5.0
9	6	4	8	5	4	6204.308	6204.311	-3.6
9	6	3	8	5	3	6204.308	6204.311	-3.6
8	7	2	7	6	2	6490.070	6490.069	1.3
8	7	1	7	6	1	6490.070	6490.069	1.3
10	6	4	9	5	4	6523.731	6523.737	-6.0
10	6	5	9	5	5	6523.731	6523.737	-6.1
9	7	3	8	6	3	6809.545	6809.546	-1.1
9	7	2	8	6	2	6809.545	6809.546	-1.1
11	6	6	10	5	6	6843.131	6843.119	11.9
11	6	5	10	5	5	6843.131	6843.118	12.2
8	8	0	7	7	0	7095.245	7095.252	-6.8
8	8	1	7	7	1	7095.245	7095.252	-6.8
10	7	4	9	6	4	7129.009	7129.008	0.7
10	7	3	9	6	3	7129.009	7129.008	0.7
12	6	7	11	5	7	7162.436	7162.443	-7.2
12	6	6	11	5	6	7162.436	7162.442	-6.3
9	8	1	8	7	1	7414.737	7414.735	2.9
9	8	2	8	7	2	7414.737	7414.735	2.9

J'	K_a'	K_c'	J''	K_a''	K_c''	ν_{obs}/MHz	ν_{calc}/MHz	$\Delta\nu$/kHz
11	7	5	10	6	5	7448.457	7448.447	10.6
11	7	4	10	6	4	7448.457	7448.447	10.6
13	6	8	12	5	8	7481.696	7481.696	-0.3
13	6	7	12	5	7	7481.696	7481.694	1.8
10	8	3	9	7	3	7734.201	7734.208	-6.9
10	8	2	9	7	2	7734.201	7734.208	-6.9
12	7	6	11	6	6	7767.853	7767.855	-1.8
12	7	5	11	6	5	7767.853	7767.855	-1.8
14	6	9	13	5	9	7800.858	7800.860	-1.5
14	6	8	13	5	8	7800.858	7800.855	3.1

Table S14: Rotational transitions for the ^{208}Pb^{20}Ne isotopologue of the assigned TEL-TFO-Ne trimer.

J'	K_a'	K_c'	J''	K_a''	K_c''	ν_{obs}/MHz	ν_{calc}/MHz	$\Delta\nu$/kHz
9	5	4	9	4	6	2787.071	2787.082	-11.0
9	5	5	9	4	5	2787.071	2787.072	-1.2
7	5	2	7	4	4	2787.591	2787.596	-4.8
7	5	3	7	4	3	2787.591	2787.594	-3.6
6	5	2	6	4	2	2787.737	2787.739	-1.9
6	5	1	6	4	3	2787.737	2787.740	-2.2
5	5	0	5	4	2	2787.823	2787.831	-8.2
5	5	1	5	4	1	2787.823	2787.831	-8.1
6	2	4	5	1	4	2821.124	2821.127	-3.3
12	1	11	11	2	9	3101.629	3101.630	-0.5

J'	K_a'	K_c'	J''	K_a''	K_c''	ν_{obs}/MHz	ν_{calc}/MHz	$\Delta\nu$/kHz
7	2	5	6	1	5	3129.608	3129.610	-2.7
5	3	2	4	2	2	3158.734	3158.734	-0.3
11	6	5	11	5	7	3406.466	3406.469	-2.5
11	6	6	11	5	6	3406.466	3406.468	-2.1
10	6	5	10	5	5	3406.756	3406.762	-5.4
10	6	4	10	5	6	3406.756	3406.762	-5.5
9	6	3	9	5	5	3406.981	3406.984	-2.3
9	6	4	9	5	4	3406.981	3406.984	-2.2
8	6	2	8	5	4	3407.139	3407.146	-7.1
8	6	3	8	5	3	3407.139	3407.146	-7.1
7	6	1	7	5	3	3407.265	3407.262	2.9
7	6	2	7	5	2	3407.265	3407.262	2.9
6	6	1	6	5	1	3407.350	3407.340	9.7
6	6	0	6	5	2	3407.350	3407.340	9.7
8	2	6	7	1	6	3437.641	3437.640	1.6
4	4	1	3	3	1	3456.979	3456.979	0.6
4	4	0	3	3	0	3456.979	3456.978	1.5
6	3	3	5	2	3	3479.678	3479.677	0.9
9	2	7	8	1	7	3745.949	3745.946	2.5
14	1	13	13	2	11	3762.414	3762.413	1.4
5	4	2	4	3	2	3779.116	3779.118	-2.2
5	4	1	4	3	1	3779.116	3779.112	4.3
7	3	4	6	2	4	3799.700	3799.698	1.9
19	3	17	18	4	15	3963.748	3963.752	-3.9
20	7	13	20	6	15	4021.171	4021.178	-7.5
20	7	14	20	6	14	4021.171	4021.175	-3.8

J'	K_a'	K_c'	J''	K_a''	K_c''	ν_{obs}/MHz	ν_{calc}/MHz	$\Delta\nu$/kHz
19	7	12	19	6	14	4022.219	4022.212	6.9
19	7	13	19	6	13	4022.219	4022.210	8.8
17	7	11	17	6	11	4023.860	4023.855	4.9
17	7	10	17	6	12	4023.860	4023.856	4.4
16	7	9	16	6	11	4024.499	4024.494	5.2
16	7	10	16	6	10	4024.499	4024.494	5.5
14	7	8	14	6	8	4025.469	4025.468	1.0
14	7	7	14	6	9	4025.469	4025.468	1.0
13	7	7	13	6	7	4025.830	4025.827	3.0
13	7	6	13	6	8	4025.830	4025.827	3.0
12	7	6	12	6	6	4026.118	4026.117	0.9
12	7	5	12	6	7	4026.118	4026.117	0.9
11	7	5	11	6	5	4026.348	4026.346	1.6
11	7	4	11	6	6	4026.348	4026.346	1.6
10	7	4	10	6	4	4026.527	4026.524	3.1
10	7	3	10	6	5	4026.527	4026.524	3.1
9	7	2	9	6	4	4026.660	4026.659	0.4
9	7	3	9	6	3	4026.660	4026.659	0.4
8	7	2	8	6	2	4026.760	4026.760	0.8
8	7	1	8	6	3	4026.760	4026.760	0.8
7	7	0	7	6	2	4026.840	4026.831	8.4
7	7	1	7	6	1	4026.840	4026.831	8.4
10	2	8	9	1	8	4055.298	4055.297	0.5
11	2	9	10	1	9	4366.467	4366.466	1.0
5	5	0	4	4	0	4398.643	4398.643	-0.1
5	5	1	4	4	1	4398.643	4398.643	-0.1

J'	K_a'	K_c'	J''	K_a''	K_c''	ν_{obs}/MHz	ν_{calc}/MHz	$\Delta\nu$/kHz
9	3	6	8	2	6	4435.562	4435.563	-1.4
15	8	7	15	7	9	4645.153	4645.154	-1.4
15	8	8	15	7	8	4645.153	4645.154	-1.4
14	8	7	14	7	7	4645.438	4645.443	-5.6
14	8	6	14	7	8	4645.438	4645.443	-5.6
13	8	5	13	7	7	4645.679	4645.680	-1.3
13	8	6	13	7	6	4645.679	4645.680	-1.3
12	8	5	12	7	5	4645.868	4645.871	-3.9
12	8	4	12	7	6	4645.868	4645.871	-3.9
11	8	4	11	7	4	4646.021	4646.024	-2.4
11	8	3	11	7	5	4646.021	4646.024	-2.4
10	8	3	10	7	3	4646.140	4646.143	-2.7
10	8	2	10	7	4	4646.140	4646.143	-2.7
9	8	2	9	7	2	4646.233	4646.234	-1.4
9	8	1	9	7	3	4646.233	4646.234	-1.4
8	8	1	8	7	1	4646.307	4646.302	5.0
8	8	0	8	7	2	4646.307	4646.302	5.0
6	5	2	5	4	2	4720.792	4720.781	11.0
6	5	1	5	4	1	4720.792	4720.781	11.1
7	5	3	6	4	3	5042.908	5042.904	3.1
7	5	2	6	4	2	5042.908	5042.904	3.5
19	1	18	18	2	16	5316.746	5316.742	3.4
14	2	12	13	1	12	5318.137	5318.139	-1.8
6	6	0	5	5	0	5340.293	5340.290	2.3
6	6	1	5	5	1	5340.293	5340.290	2.3
10	4	6	9	3	6	5388.415	5388.417	-2.1

J'	$K_a{}'$	$K_c{}'$	J''	$K_a{}''$	$K_c{}''$	ν_{obs}/MHz	ν_{calc}/MHz	$\Delta\nu$/kHz
10	4	7	9	3	7	5389.260	5389.264	-4.3
15	2	13	14	1	13	5643.557	5643.555	2.4
7	6	2	6	5	2	5662.428	5662.427	1.8
7	6	1	6	5	1	5662.428	5662.427	1.8
13	3	10	12	2	10	5681.104	5681.104	0.1
9	5	4	8	4	4	5687.060	5687.056	4.3
9	5	5	8	4	5	5687.060	5687.059	0.6
11	4	7	10	3	7	5709.571	5709.571	0.1
11	4	8	10	3	8	5711.138	5711.134	3.9
12	10	2	12	9	4	5885.025	5885.025	0.3
12	10	3	12	9	3	5885.025	5885.025	0.3
11	10	2	11	9	2	5885.113	5885.108	4.7
11	10	1	11	9	3	5885.113	5885.108	4.7
16	2	14	15	1	14	5973.974	5973.968	6.4
8	6	3	7	5	3	5984.550	5984.552	-2.6
8	6	2	7	5	2	5984.550	5984.552	-2.6
14	3	11	13	2	11	5985.834	5985.826	7.9
10	5	5	9	4	5	6009.056	6009.053	3.1
10	5	6	9	4	6	6009.056	6009.063	-6.5
12	4	8	11	3	8	6030.250	6030.251	-1.2
12	4	9	11	3	9	6032.962	6032.967	-5.2
7	7	1	6	6	1	6281.921	6281.918	3.3
7	7	0	6	6	0	6281.921	6281.918	3.3
15	3	12	14	2	12	6287.963	6287.968	-5.2
9	6	4	8	5	4	6306.658	6306.660	-2.2
9	6	3	8	5	3	6306.658	6306.660	-2.1

J'	$K_a{}'$	$K_c{}'$	J''	$K_a{}''$	$K_c{}''$	ν_{obs}/MHz	ν_{calc}/MHz	$\Delta\nu$/kHz
17	2	15	16	1	15	6309.826	6309.827	-0.9
11	5	7	10	4	7	6330.985	6330.995	-9.8
11	5	6	10	4	6	6330.985	6330.973	12.4
13	4	9	12	3	9	6350.310	6350.305	5.3
13	4	10	12	3	10	6354.788	6354.793	-5.4
16	3	13	15	2	13	6587.859	6587.861	-1.7
8	7	2	7	6	2	6604.045	6604.050	-4.6
8	7	1	7	6	1	6604.045	6604.050	-4.6
10	6	4	9	5	4	6628.740	6628.743	-3.2
10	6	5	9	5	5	6628.740	6628.743	-3.3
14	4	10	13	3	10	6669.546	6669.548	-1.8
14	4	11	13	3	11	6676.663	6676.658	4.2
17	3	14	16	2	14	6885.996	6886.000	-4.0
9	7	3	8	6	3	6926.175	6926.173	1.5
9	7	2	8	6	2	6926.175	6926.173	1.5
11	6	6	10	5	6	6950.794	6950.791	2.9
11	6	5	10	5	5	6950.794	6950.791	3.0
13	5	8	12	4	8	6974.488	6974.479	8.5
13	5	9	12	4	9	6974.568	6974.574	-5.9
15	4	12	14	3	12	6998.623	6998.627	-4.6
19	2	17	18	1	17	6999.454	6999.454	-0.5
18	3	15	17	2	15	7183.032	7183.030	1.5
8	8	1	7	7	1	7223.520	7223.521	-1.1
8	8	0	7	7	0	7223.520	7223.521	-1.1
10	7	3	9	6	3	7248.280	7248.284	-3.1
10	7	4	9	6	4	7248.280	7248.284	-3.1

J'	K_a'	K_c'	J''	K_a''	K_c''	ν_{obs}/MHz	ν_{calc}/MHz	$\Delta\nu$/kHz
12	6	7	11	5	7	7272.790	7272.795	-5.0
12	6	6	11	5	6	7272.790	7272.795	-4.6
14	5	9	13	4	9	7296.012	7296.003	9.3
16	4	12	15	3	12	7304.708	7304.707	1.6
16	4	13	15	3	13	7320.780	7320.783	-2.9
9	8	2	8	7	2	7545.646	7545.648	-1.1
9	8	1	8	7	1	7545.646	7545.648	-1.1
11	7	4	10	6	4	7570.376	7570.376	-0.0
11	7	5	10	6	5	7570.376	7570.376	-0.0
13	6	7	12	5	7	7594.745	7594.741	3.4
13	6	8	12	5	8	7594.745	7594.742	2.4
10	8	3	9	7	3	7867.759	7867.767	-8.2
10	8	2	9	7	2	7867.759	7867.767	-8.2
12	7	5	11	6	5	7892.440	7892.443	-3.7
12	7	6	11	6	6	7892.440	7892.443	-3.7
14	6	9	13	5	9	7916.620	7916.620	-0.7
14	6	8	13	5	8	7916.620	7916.618	1.7
9	9	0	8	8	0	8165.096	8165.096	-0.1
9	9	1	8	8	1	8165.096	8165.096	-0.1

Table S15: Rotational transitions for the ^{208}Pb^{22}Ne isotopologue of the assigned TEL-TFO-Ne trimer.

J'	K_a'	K_c'	J''	K_a''	K_c''	ν_{obs}/MHz	ν_{calc}/MHz	$\Delta\nu$/kHz
5	3	2	4	2	2	3109.007	3109.006	0.6

J'	$K_a{}'$	$K_c{}'$	J''	$K_a{}''$	$K_c{}''$	ν_{obs}/MHz	ν_{calc}/MHz	$\Delta\nu$/kHz
15	6	9	15	5	11	3325.736	3325.741	-5.4
15	6	10	15	5	10	3325.736	3325.721	14.8
14	6	9	14	5	9	3326.677	3326.685	-7.7
14	6	8	14	5	10	3326.677	3326.695	-17.8
11	6	6	11	5	6	3328.594	3328.587	6.8
11	6	5	11	5	7	3328.594	3328.588	5.9
10	6	5	10	5	5	3328.977	3328.968	8.6
10	6	4	10	5	6	3328.977	3328.969	8.2
9	6	4	9	5	4	3329.250	3329.256	-6.5
9	6	3	9	5	5	3329.250	3329.256	-6.6
8	6	2	8	5	4	3329.481	3329.467	13.7
8	6	3	8	5	3	3329.481	3329.467	13.8
7	6	2	7	5	2	3329.605	3329.617	-11.7
7	6	1	7	5	3	3329.605	3329.617	-11.7
8	2	6	7	1	6	3388.410	3388.412	-2.4
4	4	1	3	3	1	3396.232	3396.236	-4.5
4	4	0	3	3	0	3396.232	3396.235	-3.1
7	3	4	6	2	4	3743.323	3743.327	-4.4
10	2	8	9	1	8	4001.318	4001.316	1.4
8	3	5	7	2	5	4058.285	4058.281	3.4
5	5	0	4	4	0	4320.951	4320.949	2.5
5	5	1	4	4	1	4320.951	4320.949	2.5
9	3	6	8	2	6	4371.174	4371.174	0.2
6	5	2	5	4	2	4640.241	4640.245	-4.1
6	5	1	5	4	1	4640.241	4640.245	-4.0
10	3	7	9	2	7	4681.581	4681.587	-5.1

J'	K_a'	K_c'	J''	K_a''	K_c''	ν_{obs}/MHz	ν_{calc}/MHz	$\Delta\nu$/kHz
13	2	11	12	1	11	4942.768	4942.768	0.1
7	5	2	6	4	2	4959.521	4959.522	-0.5
7	5	3	6	4	3	4959.521	4959.522	-1.1
11	3	8	10	2	8	4989.183	4989.179	4.4
9	4	5	8	3	5	4991.704	4991.698	6.5
9	4	6	8	3	6	4992.328	4992.332	-5.0
6	6	0	5	5	0	5245.643	5245.643	0.2
6	6	1	5	5	1	5245.643	5245.643	0.2
8	5	3	7	4	3	5278.765	5278.764	0.5
8	5	4	7	4	4	5278.765	5278.766	-1.6
10	4	6	9	3	6	5310.153	5310.130	23.1
10	4	7	9	3	7	5311.394	5311.391	3.1
7	6	2	6	5	2	5564.935	5564.937	-2.6
7	6	1	6	5	1	5564.935	5564.937	-2.6
11	4	7	10	3	7	5628.081	5628.082	-1.2
11	4	8	10	3	8	5630.405	5630.407	-2.0
10	5	5	9	4	5	5917.081	5917.071	10.7
10	5	6	9	4	6	5917.081	5917.087	-5.7
12	4	8	11	3	8	5945.375	5945.379	-4.0
7	7	0	6	6	0	6170.314	6170.315	-1.0
7	7	1	6	6	1	6170.314	6170.315	-1.0
9	6	4	8	5	4	6203.472	6203.478	-6.5
9	6	3	8	5	3	6203.472	6203.478	-6.5
11	5	6	10	4	6	6236.068	6236.085	-16.9
13	4	9	12	3	9	6261.804	6261.807	-2.9
13	4	10	12	3	10	6268.455	6268.458	-2.8

J'	$K_a{}'$	$K_c{}'$	J''	$K_a{}''$	$K_c{}''$	ν_{obs}/MHz	ν_{calc}/MHz	$\Delta\nu$/kHz
8	7	2	7	6	2	6489.609	6489.605	3.2
8	7	1	7	6	1	6489.609	6489.605	3.2
10	6	4	9	5	4	6522.708	6522.706	2.1
10	6	5	9	5	5	6522.708	6522.706	2.0
14	4	10	13	3	10	6577.109	6577.108	0.6
14	4	11	13	3	11	6587.624	6587.622	2.2
9	7	3	8	6	3	6808.888	6808.885	3.1
9	7	2	8	6	2	6808.888	6808.885	3.1
11	6	5	10	5	5	6841.894	6841.891	3.0
11	6	6	10	5	6	6841.894	6841.891	2.7
12	6	6	11	5	6	7161.014	7161.018	-4.0
12	6	7	11	5	7	7161.014	7161.019	-4.9
9	8	1	8	7	1	7414.248	7414.245	3.1
9	8	2	8	7	2	7414.248	7414.245	3.1
11	7	5	10	6	5	7447.393	7447.390	2.5
11	7	4	10	6	4	7447.393	7447.390	2.5
13	6	7	12	5	7	7480.070	7480.073	-3.1
13	6	8	12	5	8	7480.070	7480.075	-5.2
10	8	2	9	7	2	7733.522	7733.521	1.1
10	8	3	9	7	3	7733.522	7733.521	1.1
12	7	5	11	6	5	7766.606	7766.602	4.3
12	7	6	11	6	6	7766.606	7766.602	4.3
14	6	9	13	5	9	7799.045	7799.043	1.7
14	6	8	13	5	8	7799.045	7799.038	6.3
9	9	1	8	8	1	8019.571	8019.576	-4.9
9	9	0	8	8	0	8019.571	8019.576	-4.9
